# The Inhibition of B7H3 by 2-HG Accumulation Is Associated With Downregulation of VEGFA in IDH Mutated Gliomas

**DOI:** 10.3389/fcell.2021.670145

**Published:** 2021-05-17

**Authors:** Mengli Zhang, Huaichao Zhang, Minjie Fu, Jingwen Zhang, Cheng Zhang, Yingying Lv, Fengfeng Fan, Jinsen Zhang, Hao Xu, Dan Ye, Hui Yang, Wei Hua, Ying Mao

**Affiliations:** ^1^Department of Neurosurgery, Huashan Hospital, Fudan University, Shanghai, China; ^2^Institutes of Biomedical Sciences, Fudan University, Shanghai, China; ^3^Key Laboratory of Metabolism and Molecular Medicine, Ministry of Education, The Molecular and Cell Biology Lab, Key Laboratory of Medical Epigenetics and Metabolism, Shanghai Medical College, Institutes of Biomedical Sciences, Fudan University, Shanghai, China; ^4^Department of General Surgery, Huashan Hospital, Fudan University, Shanghai, China; ^5^Ministry of Education Frontiers Center for Brain Science, Institute for Translational Brain Research, Shanghai Medical College, Fudan University, Shanghai, China; ^6^Institutes of Biomedical Sciences, Fudan University, Shanghai, China; ^7^State Key Laboratory of Medical Neurobiology, School of Basic Medical Sciences and Institutes of Brain Science, Fudan University, Shanghai, China; ^8^The Collaborative Innovation Center for Brain Science, Fudan University, Shanghai, China

**Keywords:** B7H3, glioma, IDH mutation, 2-HG, autophagy, VEGFA

## Abstract

B7H3 (also known as CD276) is a co-stimulator checkpoint protein of the cell surface B7 superfamily. Recently, the function beyond immune regulation of B7H3 has been widely studied. However, the expression preference and the regulation mechanism underlying B7H3 in different subtypes of gliomas is rarely understood. We show here that B7H3 expression is significantly decreased in IDH-mutated gliomas and in cultured IDH1-R132H glioma cells. Accumulation of 2-HG leads to a remarkable downregulation of B7H3 protein and the activity of IDH1-R132H mutant is responsible for B7H3 reduction in glioma cells. Inhibition of autophagy by inhibitors like leupeptin, chloroquine (CQ), and Bafilomycin A1 (Baf-A1) blocks the degradation of B7H3 in glioma cells. In the meantime, the autophagy flux is more active with higher LC3B-II and lower p62 in IDH1-R132H glioma cells than in IDH1-WT cells. Furthermore, sequence alignment analysis reveals potential LC3-interacting region (LIR) motifs “F-V-S/N-I/V” in B7H3. Moreover, B7H3 interacts with p62 and CQ treatment significantly enhances this interaction. Additionally, we find that *B7H3* is positively correlated with *VEGFA* and *MMP2* by bioinformatics analysis in gliomas. B7H3 and VEGFA are decreased in IDH-mutated gliomas and further reduced in 2-HG^high^ gliomas compared to 2-HG^low^ glioma sections by IHC staining. Our study demonstrates that B7H3 is preferentially overexpressed in IDH wild-type gliomas and could serve as a potential theranostic target for the precise treatment of glioma patients with wild-type IDH.

## Introduction

Gliomas are the most common and aggressive primary malignant brain tumors, which are highly lethal (Stupp et al., 2005). Recent molecular profiling has brought a comprehensive diagnosis of glioma based on certain biomarkers (Louis et al., 2016), which are becoming essential for precise therapy. Anti-VEGFA drugs could benefit proneural glioblastoma (GBM) patients (Gilbert et al., 2014), and anti-PD-1 drugs seem effective for BRAF mutated GBM (Zhao et al., 2019). ONC201 might be a good candidate for treating H3 K27M-mutant diffuse gliomas (Arrillaga-Romany et al., 2017; Chi et al., 2019; Hall et al., 2019). Thus, the molecular subclassification of gliomas potentially improves therapeutic benefit in patients.

B7 family are known as immune checkpoint proteins, and antibody against PD-L1 (aka B7H1) has achieved survival benefit in many solid tumors (Sznol and Chen, 2013; Wolchok et al., 2013). In our previous report, B7H3 accumulated the most in GBM than other B7 family members (Zhang et al., 2019). Early reports focused on the expression and immune regulation of B7H3 on dendritic cells (DCs), monocytes (Chapoval et al., 2001), T cell inhibition (Pardoll, 2012; Chen et al., 2013; Vigdorovich et al., 2013), and also NK cells and invasive phenotype in GBM (Lemke et al., 2012). However, the lack of receptor hindered further immune investigation of B7H3 on the immune regulation. The functions beyond immune regulation of B7H3 have been widely studied (Flem-Karlsen et al., 2018), and we found that B7H3 was correlated with the maintenance of glioma self-renewing cell (GSCs) *via* the TGF-β pathway and MYC activation (Zhang et al., 2019). Both the immune and non-immune functions contributed to the tries of targeting B7H3 in gliomas (Khan et al., 2020). B7H3-redirected CAR T cells could release effector cytokines, IFN-γ and IL-2, and target GBM cell lines and patient-derived neurospheres *in vitro* and *in vivo* (Nehama et al., 2019), as also reported in other tumor-related xenograft models including medulloblastoma, Ewing sarcoma, osteosarcoma (Majzner et al., 2019), AML, and melanoma (Zhang et al., 2020). In addition, pyrrolobenzodiazepine (PBD)-conjugated B7H3 ADCs (antibody-drug conjugate) could effectively kill both the cancer cells and tumor vasculature in MC38 cells and in mouse models (Seaman et al., 2017), indicating a potential productive relationship with angiogenesis.

It has been reported that B7H3 showed significant correlation with IDH1 level in colorectal cancer (CRC), and the co-expression could predict a poor prognosis (Wu et al., 2018). Moreover, the heterogeneous expression of B7H3 in gliomas and other tumors were also observed (Zhang et al., 2019), and B7H3 seems to favor its expression in middle line gliomas (Zhou et al., 2013). In the present study, we found the protein expression of B7H3 is significantly decreased in IDH-mutated gliomas compared to the IDH wild-type gliomas, which is due to 2-HG accumulation and its reduction is likely to be mediated by active autophagy degradation pathway. Meanwhile, the downregulation of VEGFA in IDH-mutated gliomas is also associated with low protein level of B7H3 and high 2-HG level. In summary, our results demonstrate that B7H3 is preferentially overexpressed in *IDH* wild-type gliomas and could potentially serve as a theranostic indicator for precise glioma treatment.

## Materials and Methods

### Clinical Specimen Collection

The collection of human glioma samples was approved by the ethics committee of Huashan Hospital, Fudan University. Human glioma tissues were collected from the Neurological Surgery Department of Huashan Hospital, Fudan University between January 2010 and July 2020. Informed consents were obtained from all patients. Glioma samples were obtained during surgical resection, snap frozen by liquid nitrogen for intraoperative frozen pathology and fresh samples were undergone western blotting and immunohistochemistry. Preliminary judgment of neoplasm tissue was dependent on frozen pathology and formal clinical classification and grading of these samples was performed by neuropathologists according to the 2016 WHO Classification of Tumors of the Central Nervous System (Louis et al., 2016).

### Antibodies

Antibodies specific to β-ACTIN (GeneScript, mouse monoclonal antibody, A00702, 1:10,000), B7H3 (R&D Systems, Polyclonal Goat IgG, AF1027, 1:1,000), IDH1 (Abcam, rabbit monoclonal antibody, ab172964, 1:1,000), Flag (Sigma, mouse monoclonal antibody, F9291, 1:3,000), β-Catenin (Cell Signaling Technology, rabbit monoclonal antibody, 8480, 1:1,000), p27 (Cell Signaling Technology, rabbit polyclonal antibody, 2552, 1:1,000), LC3B (Cell Signaling Technology, rabbit monoclonal antibody, 3868, 1:1,000), p62 (Cell Signaling Technology, mouse monoclonal antibody, 88588, 1:1,000), p21 (santa cruz, mouse monoclonal antibody, sc-271532, 1:1,000), VEGFA (proteintech, mouse monoclonal antibody, 66828-1-Ig, 1:1,000), STAT3 (Cell Signaling Technology, rabbit monoclonal antibody, 12640, 1:1,000), phospho-STAT3 (Cell Signaling Technology, rabbit monoclonal antibody, 9145, 1:2,000), ERK1/2 (Cell Signaling Technology, rabbit monoclonal antibody, 4695, 1:1,000), phospho-ERK1/2 (Cell Signaling Technology, rabbit monoclonal antibody, 4370, 1:2,000),IDH1-R132H (Dianova, mouse monoclonal antibody, DIA-H09, 1:1,000), p53 (santa cruz, mouse monoclonal antibody, sc-126, 1:1,000), c-Myc (Cell Signaling Technology, rabbit monoclonal antibody, 5605, 1:1,000), SMAD1 (Cell Signaling Technology, rabbit monoclonal antibody, 6944, 1:1,000), phospho-SMAD1 (Cell Signaling Technology, rabbit monoclonal antibody, 5753, 1:1,000), SMAD4 (Cell Signaling Technology, rabbit monoclonal antibody, 38454, 1:1,000), SMAD6 (Abcam, rabbit polyclonal antibody, ab80049, 1:1,000), Phospho-NF-κB p65 (Ser536) (Cell Signaling Technology, rabbit monoclonal antibody, 3033, 1:1,000), and NF-κB p65 (Cell Signaling Technology, rabbit monoclonal antibody, 8242, 1:1,000) were purchased commercially. Secondary antibodies for polyclonal goat anti-mouse IgG light chain and monoclonal mouse anti-rabbit IgG light chain (Jackson ImmunoResearch, 115-035-174 and 211-032-171, respectively, both 1:3,000), and polyclonal donkey anti-goat IgG (H&L) (Genscript, A00178, 1:3,000) were also purchased commercially.

### Plasmid Construction

The cDNA encoding full-length human IDH1 was cloned into Flag-tagged pCDH-CMV-MCS-EF1-puro vector. Flag-IDH1-R132H and Flag-IDH1-R132H/T77A (a double mutant reported to eliminate the D-2-HG producing activity; Ma et al., 2015; Wang et al., 2015) point mutants were generated from Flag-IDH1 by using the ClonExpress^®^ II One Step Cloning kit (Vazyme, c112-02).

### Cell Culture, Transfection, and Treatment

Human glioma cells U87 and U251 (Obtained from ATCC in September 2017, ATCC^®^ HTB-14, and SGST.CN in November 2019, TCHu 58, respectively) were cultured in Dulbecco’s Modified Eagle’s Medium (DMEM, Gibco) supplemented with 5% fetal bovine serum (Gibco), 100 units/mL penicillin and 100 μg/mL streptomycin, in 5% CO_2_ atmosphere at 37°C. Cell transfection was carried out by polyethyleneimine (PEI, Sigma-Aldrich). For 2-HG treatment, 1 or 2 mM (2R)-Octyl-α-hydroxyglutarate (D-2-HG, MCE, HY-103641) was added into U87 and U251 cells and incubated for the indicated time. For CHX (Selleck, S7418), MG132 (Selleck, S2619), Bortezomib (BTZ, Selleck, S1013), Pevonedistat (MLN4924, Selleck, S7109), leupeptin (Selleck, S7380), chloroquine (CQ, Selleck, S6999), and Bafilomycin A1 (Baf-A1, Selleck, S1413) treatments, the corresponding drugs were added in U87 or U251 cells for the indicated time.

### Western Blotting and Immunoprecipitation

For human glioma tissue lysate, glioma samples were homogenized in ice-cold 0.3% NP-40 buffer containing 50 mM Tris-HCl (pH 7.5), 300 mM NaCl, 0.3% NP-40, 1 mM Na_3_VO_4_ and protease inhibitor cocktail (Biotool) with the Tissuelyser-24 (Shanghai JingXin). Glioma homogenates were lysed with rotation at 4°C for 15 min and centrifugated at 13,000 rpm for 15 min at 4°C. The supernatant was collected, lysed in 1 × SDS sample buffer and denatured by heating on 99°C for 15 min, followed by direct western blot analysis. For whole cell lysate, cells were lysed in 1 × SDS sample buffer and denatured by heating on 99°C for 15 min and then subjected to SDS-PAGE and immunoblotting according to the standard methods. For Flag immunoprecipitation, cells were lysed in ice-cold 0.3% NP-40 buffer containing 50 mM Tris-HCl (pH 7.5), 150 mM NaCl, 1 mM Na_3_VO_4_ and protease inhibitor cocktail (Biotool). Cell lysate was incubated with anti-Flag M2 affinity beads (Sigma, A2220) for 3 h at 4°C, washed three times with ice-cold 0.4% NP-40 buffer, and analyzed by SDS-PAGE. Immunoblotting was carried out according to standard methods.

### Metabolite Extraction and LC-MS/MS for 2-HG Quantification

Intracellular 2-HG level of targeted cells were measured by using liquid chromatography-mass spectrometry (LC-MS/MS) (Zeng et al., 2013). Briefly, cells for the indicated time were washed with ice-cold PBS twice and harvested in 80% (v/v) pre-cold (−80°C) methanol overnight or rotating at 4°C for 90 min and centrifuged at 4°C at 14,000 rpm for 30 min. The supernatant was lyophilized and then was resuspended in 50 μL of reconstitution fluid (ACN: H_2_O = 5:95, v/v), mixed well and centrifuged for 15 min at 14,000 rpm at 4°C. Aliquot 40 μL of supernatant was then subjected to LC-MS/MS (Waters ACQUITY UPLC^®^ I-Class system coupled with Xevo^®^ TQ-XS mass spectrometry). Separation of the 2-HG metabolite was achieved in Waters ACQUITY UPLC HSS T3 column (2.1 mm × 100 mm × 1.8 μm). The elution solvents consisted of A (0.2% formic acid in water, containing 10 mM ammonium acetate) and B (0.2% formic acid in acetonitrile). The elution gradient was set as follows: 100% A (0.0–0.1 min), 100–98% A (0.1–2.0 min), 98–40% (2.0–3.5 min), 40–5% (3.5–4.0 min), 5–5% (4.0–5.0 min), 5–100% A (5.0–5.01 min), 100–100% A (5.01–7 min). The column temperature was set to be 45°C and the flow rate was 0.4 mL/min. The data were acquired in both ESI positive and negative modes. The Capillary was 3.0 kV for positive mode and 2.53 kV for negative mode and the Cone was 20 V. The Desolvation Temp and Source Temp were set to 600 and 150°C, respectively. The Analyst Software MassLynx v4.2 was used for analysis.

### Sequence Alignment and Motif Logo Analysis

Sequences of human (*Homo sapiens*), mouse (*Mus musculus*), rat (*Rattus norvegicus*), and Xenla (X*enopus laevis*) were selected, downloaded from Uniprot^1^, and then aligned by Jalview^2^. The motif logo was also conducted by Jalview (see text footnote 2).

### Gene Expression and Correlation Analysis

TCGA^3^ and CGGA^4^ datasets were downloaded and used to analyzed the mRNA expression of genes of interest. Gene expression correlation analysis was also performed on the TCGA and CGGA expression data. Pearson correlation and linear regression were conducted with R version 3.6.1^5^.

### Immunohistochemistry (IHC) Analysis

According to the manufacturer’s protocol, the specimens were immunostained with anti-B7H3 (R&D Systems, Polyclonal Goat IgG, AF1027, 1:200), anti-VEGFA (proteintech, rabbit polyclonal antibody, 19003-1-AP, 1:100) and anti-IDH1-R132H (Dianova, mouse monoclonal antibody, DIA-H09, 1:100) antibodies. Immune reactive score (IRS) was calculated as described (Lemke et al., 2012) and used for statistical analysis. Images were obtained by scanning IHC slides with PORTABLE DIGITAL MICROSCOPE AND SLIDE SCANNER (Ocus, Grundium, Finland).

### Statistics

All the statistical analyses were performed with a two-tailed unpaired Student’s *t*-test. All data shown represent the results obtained from at least triplicated independent experiments with standard deviations of the mean (mean ± SD). The values of *p* < 0.05 were considered statistically significant.

All relevant materials will be available upon request to interested researchers.

## Results

### B7H3 Is Down-Regulated in IDH-Mutated Gliomas

**FIGURE 1:**
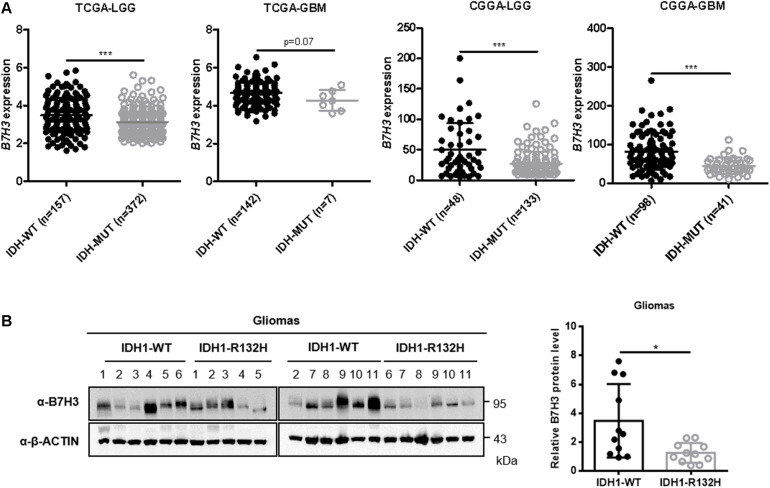
B7H3 is downregulated in IDH-mutated gliomas. **(A)** *B7H3* mRNA expression is significantly decreased in IDH-mutated gliomas according to the TCGA and CGGA datasets. Gliomas from the TCGA and CGGA databases was separated into lower-grade glioma (LGG) and GBM subgroups, respectively. In the TCGA-LGG subtype, *n* = 157 in IDH-WT and *n* = 372 in IDH-MUT groups, respectively, in the TCGA-GBM subtype, *n* = 142 in IDH-WT and *n* = 7 in IDH-MUT groups, respectively, in the CGGA-LGG subtype, *n* = 48 in IDH-WT and *n* = 133 in IDH-MUT groups, respectively, and in the CGGA-GBM subtype, *n* = 98 in IDH-WT and *n* = 41 in IDH-MUT groups, respectively. MUT = mutant. ***denotes the *P* < 0.001 for the indicated comparisons. **(B)** B7H3 protein level is significantly reduced in IDH1-R132H glioma specimens. IDH1-WT and IDH1-R132H fresh human glioma specimens (*n* = 11 per group) were collected, and B7H3 protein level was detected by immunoblotting using the indicated antibodies (left). Quantification of the protein level of B7H3 was shown on the right. *denotes the *P* < 0.05 for the indicated comparison.

To investigate the expression pattern of B7H3 in gliomas, we first analyzed its mRNA level according to the Cancer Genome Atlas (TCGA) and Chinese Glioma Genome Atlas (CGGA) database. We found that the mRNA level of *B7H3* was significantly decreased in IDH-mutated lower grade gliomas (LGG) (*n* = 372) compared to that in IDH wild-type (WT) LGG (*n* = 157) in TCGA dataset (*p* < 0.001, Figure 1A). Although the difference failed to reach statistical significance, the expression of *B7H3* tended to be lower in IDH-mutated GBM (n = 7) compared to that in IDH-WT GBM (*n* = 142) according to TCGA dataset analysis (*p* = 0.07, Figure 1A). Likewise, we found that *B7H3* was dramatically declined in both IDH-mutated LGG (*n* = 133) and GBM (*n* = 41) compared with that in the corresponding IDH-WT LGG (*n* = 48, *p* < 0.001) and GBM (*n* = 98, *p* < 0.001) in CGGA database (Figure 1A). Next, we examined the protein level of B7H3 in fresh glioma tissues and found that the protein expression of B7H3 was significantly reduced in IDH1-R132H gliomas (*n* = 11) compared to that in IDH1-WT gliomas (*n* = 11, *p* < 0.05, Figure 1B). These results together demonstrate that B7H3 is downregulated in IDH-mutated gliomas.

### D-2-HG Accumulation Leads to B7H3 Downregulation in U87 and U251 Cells

**FIGURE 2:**
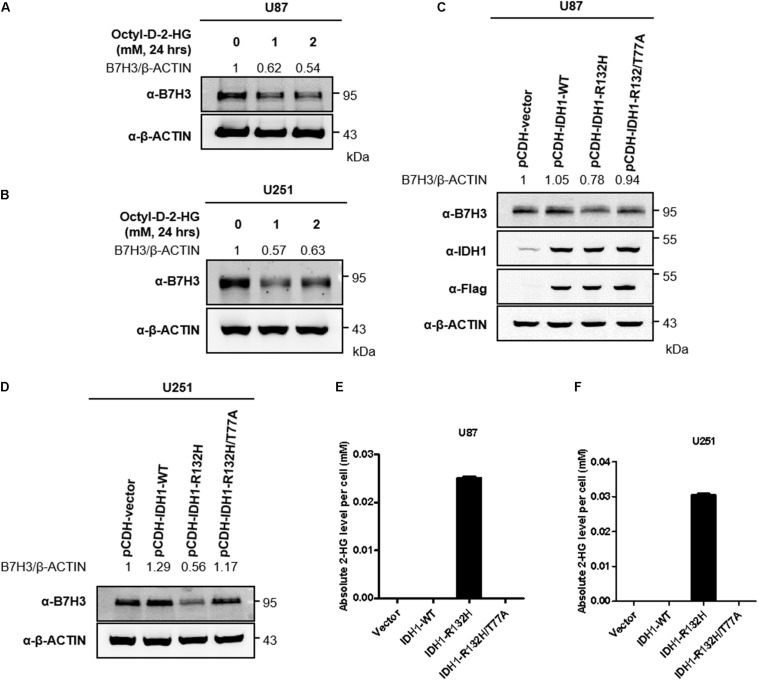
2-HG accumulation leads to B7H3 downregulation in U87 and U251 cells. **(A)** B7H3 is decreased after 2-HG treatment in U87 cell line. Cells were collected after cell-permeable octyl-D-2HG treatment for 24 h, and immunoblotting was performed with the indicated antibodies. **(B)** B7H3 is reduced after 2-HG treatment in U251 cell line. Cells were collected after cell-permeable octyl-D-2HG treatment for 24 h, and immunoblotting was performed with the indicated antibodies. **(C)** B7H3 is decreased in stable IDH1-R132H U87 cell line. Stably expressed IDH1-WT, IDH1-R132H, and IDH1-R132H/T77A U87 cells were constructed. After cell collection, immunoblotting was performed with the indicated antibodies. **(D)** B7H3 is reduced in stable IDH1-R132H U251 cell line. Stably expressed IDH1-WT, IDH1-R132H, and IDH1-R132H/T77A U251 cells were constructed. After cell collection, immunoblotting was performed with the indicated antibodies. **(E,F)** Absolute 2-HG levels in stable U87 and U251 cell lines. Vector, IDH1-WT, IDH1-R132H, and IDH1-R132H/T77A U87 **(E)** and U251 cells **(F)** were collected and subjected to LC-MS/MS for 2-HG measurement as described in section “Materials and Methods.” All experiments were performed in triplicate and representative results were shown.

Tumor-derived IDH1 and IDH2 mutations not only simultaneously lose their normal catalytic activity: the production of α-ketoglutarate (α-KG) from the convertion of isocitrate, but also gain a neomorphic enzymatic activity: the reduction of α-KG to D-2-hydroxyglutarate (D-2-HG) (Dang et al., 2009; Yan et al., 2009; Zhao et al., 2009). To determine whether D-2-HG affects the expression of B7H3, we treated a glioma cell line U87 with cell-permeable D-2-HG at 1, and 2 mM for 24 h, and found that D-2-HG significantly decreased B7H3 protein level (Supplementary Figures 1A,B and Figure 2A). As expected, B7H3 expression was also found reduced in another glioma cell line U251 after cell-permeable D-2-HG treatment (Supplementary Figures 1A,B and Figure 2B). To investigate whether downregulation of B7H3 by D-2-HG is dependent on the neomorphic enzymatic activity of IDH mutation, we stably established U87 cells expressing IDH1-WT, IDH1-R132H mutant, or IDH1-R132H/T77A double mutant (Figure 2C and Supplementary Figure 2A). The IDH1-R132H/T77A double mutant was reported to impede the catalytic activity on the production of D-2-HG (Wang et al., 2015). We found that the protein level of B7H3 was decreased in IDH1-R132H U87 cells, while was increased to the comparable level in IDH1-R132H/T77A cells compared to that in IDH1-WT U87 cells (Figure 2C). Also, the parallel results of B7H3 level were observed in stably constructed IDH1-WT, IDH1-R132H, and IDH1-R132H/T77A U251 cells (Figure 2D and Supplementary Figures 2B,C), which indicates the high level of D-2-HG is important for the degradation of B7H3. Quantitative results by LC-MS/MS analysis further showed that the levels of D-2-HG were accumulated as high as 0.025 mM and 0.03 mM in IDH1-R132H U87 and IDH1-R132H U251 cells, respectively (Figures 2E,F). Together, these findings demonstrate that D-2-HG accumulation leads to the downregulation of B7H3 in IDH-mutated glioma cells.

### Autophagy Inhibition Blocks B7H3 Degradation

**FIGURE 3:**
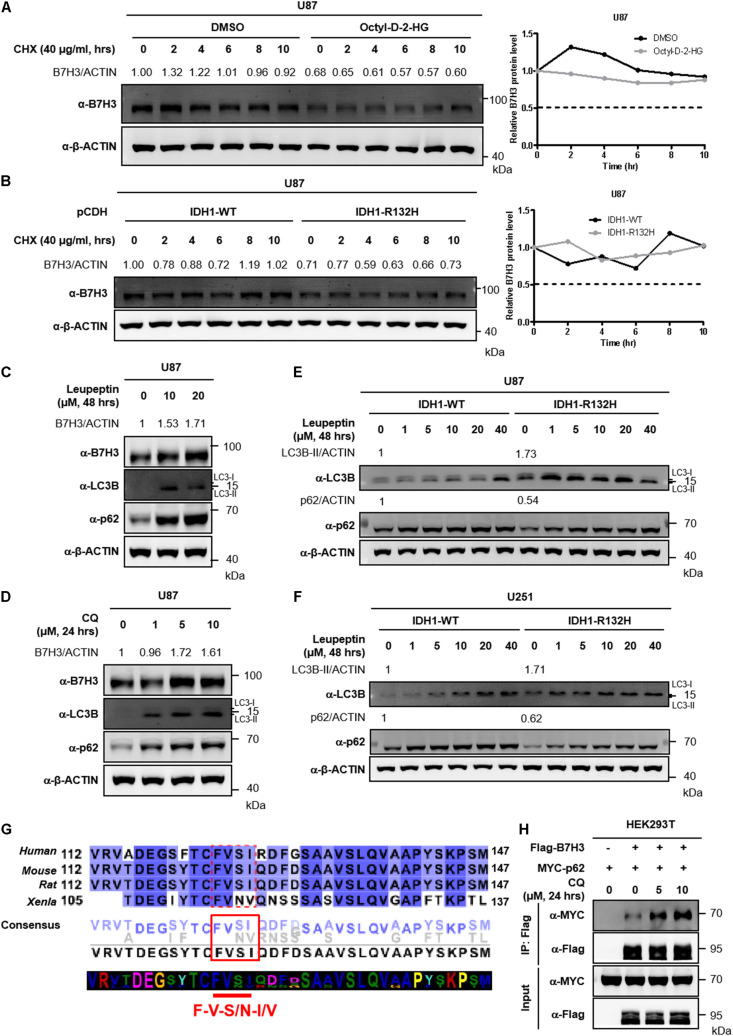
Inhibition of autophagy blocks the degradation of B7H3. **(A)** B7H3 is a stable protein in U87 cells. Cells were pretreated with DMSO or cell-permeable octyl-D-2HG and then 40 μg/mL CHX was added in the cells for the indicated time course. After cell collection, immunoblotting was performed with the indicated antibodies (left). Relative quantification of B7H3 protein level was shown on the right. **(B)** B7H3 is a stable protein in IDH1-WT U87 cells. Stably expressed IDH1-WT or IDH1-R132H U87 cells were treated with 40 μg/mL CHX for the indicated time course. Immunoblotting was performed with the indicated antibodies (left). Relative quantification of B7H3 protein level was shown on the right. **(C)** Leupeptin treatment blocks the degradation of B7H3 in U87 cells. Cells were treated with leupeptin for the indicated concentrations and time. After cell collection, immunoblotting was performed with the indicated antibodies. **(D)** CQ treatment blocks the degradation of B7H3 in U87 cells. Cells were treated with CQ for the indicated concentrations and time. After cell collection, immunoblotting was performed with the indicated antibodies. **(E,F)** Autophagy is more active in IDH1-R132H U87 and U251 cells. Stable IDH1-WT and IDH1-R132H U87 **(E)** and U251 **(F)** cells were collected for immunoblotting with the indicated antibodies. Gradient concentrations of leupeptin treating cells for 48 h were used to verify LC3B and p62 bands. **(G)** B7H3 contains a potential LIR motif. B7H3 amino acid sequences in species including *Homo sapiens, Mus musculus, Rattus norvegicus, and Xenopus laevis* were analyzed by the online tool Jalview (http://www.jalview.org/). Dotted red box, solid red box and red straight line indicate the potential LIR sequences in B7H3. **(H)** CQ treatment enhances the interaction between B7H3 and p62 in HEK293T cells. HEK293T cells were seeded in the six-well plate at a density of 0.4 × 10^6^. Flag-B7H3 was co-expressed with Myc-p62 in HEK293T cells. 24 h later after plasmid transfection, CQ was added in the cells and incubated for another 24 h. Cells were lysed and proteins were immunoprecipitated with anti-Flag M2 affinity beads. Immunoblotting was performed with the indicated antibodies. All experiments were performed in triplicate and representative results were shown.

Inhibition of total protein synthesis with cycloheximide (CHX, an agricultural fungicide that inhibits *de novo* protein synthesis) (Liu et al., 2020; Moroso et al., 2020) showed that B7H3 was a rather stable protein in U87 cells with a half-life time longer than 10 h (Figure 3A). Similarly, the half-life of B7H3 was still longer than 10 h in stable IDH1-WT U87 cells after CHX treatment (Figure 3B), re-affirming that B7H3 is a stable protein. In agreement with the findings in Figure 2, B7H3 was again found decreased in U87 cells with cell-permeable D-2-HG treatment or with stable IDH1-R132H overexpression (Figures 3A,B). However, the expression of B7H3 was not further declined with CHX treatment (Figures 3A,B), which is in accord with the stability of B7H3 observed above.

To investigate how B7H3 was degraded in IDH mutant glioma cells, we screened U87 cells and IDH1-R132H U87 cells with several inhibitors by targeting intracellular protein-degradation pathways. The results showed that treatment with the proteasome inhibitor MG132 did not elevate B7H3, but significantly increased the protein level of β-catenin (a member of Wnt/β-catenin-signaling pathways) targeted by the proteasome for degradation (Paul et al., 2019) in U87 and IDH1-R132H U87 cells (Supplementary Figures 3A, 4A). Moreover, another proteasome inhibitor BTZ, also could not accumulate B7H3 protein in U87 and IDH1-R132H U87 cells (Supplementary Figures 3B, 4B). Additionally, the neddylation inhibitor MLN4924 still could not increase B7H3 protein level in U87 and IDH1-R132H U87 cells (Supplementary Figures 3C, 4C). These results indicate that the IDH mutation- or 2-HG-induced decrease of B7H3 is mediated by a mechanism that is independent of proteasome or neddylation. Autophagy is a major mechanism for intracellular protein degradation. We treated U87 cells with leupeptin, an inhibitor of autophagy which can block lysosome-dependent protein degradation (Jeong et al., 2009; Zhao et al., 2013) and found that this treatment could cause a significant increase of B7H3 protein (Figure 3C). Alternatively, B7H3 was also accumulated after leupeptin treatment in IDH1-R132H U87 cells (Supplementary Figure 4D). Likewise, Baf-A1, another autophagy inhibitor, could lead to a significant accumulation of B7H3 protein in IDH1-R132H U87 cells, too (Supplementary Figure 4E). CQ is an FDA-approved drug used for the treatment of tumors via autophagy inhibition by blocking autophagosome fusion and degradation (Mauthe et al., 2018) and we found that B7H3 protein was significantly accumulated by treating U87 cells with CQ (Figure 3D). These findings suggest that B7H3 degradation is mediated by autophagy. We further showed that the levels of autophagy-related markers, LC3B-II and p62 (Cohen-Kaplan et al., 2016; Chen et al., 2018) were significantly higher and lower, respectively in IDH1-R132H U87 cells when compared to the IDH1-WT cells (Figure 3E). Similarly, a parallel results of LC3B-II and p62 was also observed in IDH1-R132H U251 cells when compared to that in IDH1-WT cells (Figure 3F), which suggests an activated autophagy flux in IDH1-R132H U87 and U251 cells compared to IDH1-WT cells. In general, proteins containing LC3-interacting region (LIR) motif(s) that can be recognized by and interacted with LC3 can subsequently undergo the autophagy degradation (Birgisdottir et al., 2013). The classic core consensus LIR motifs are W/F/Y-X-X-L/I/V (X represents any amino acid) (Birgisdottir et al., 2013). We then analyzed the amino acid sequences of B7H3 in species including *Homo sapiens, Mus musculus, Rattus norvegicus, and Xenopus laevis* and found that F-V-S/N-I/V sequences in B7H3 proteins were in accord with the classic motifs (Figure 3G), suggesting that B7H3 was likely to interact with LC3 via its potential LIR motifs recognized by LC3. Additionally, we conducted the co-immunoprecipitation (Co-IP) assay and found that B7H3 bound to p62 and the interaction was significantly enhanced by CQ treatment (Figure 3H). Collectively, these results indicate that autophagy is involved in B7H3 degradation and the observed decrease of B7H3 by D-2-HG or IDH mutation is most likely due to its accelerated degradation *via* the autophagy pathway.

### *B7H3* Is Positively Correlated With *VEGFA* and *MMP2* in Gliomas

**FIGURE 4:**
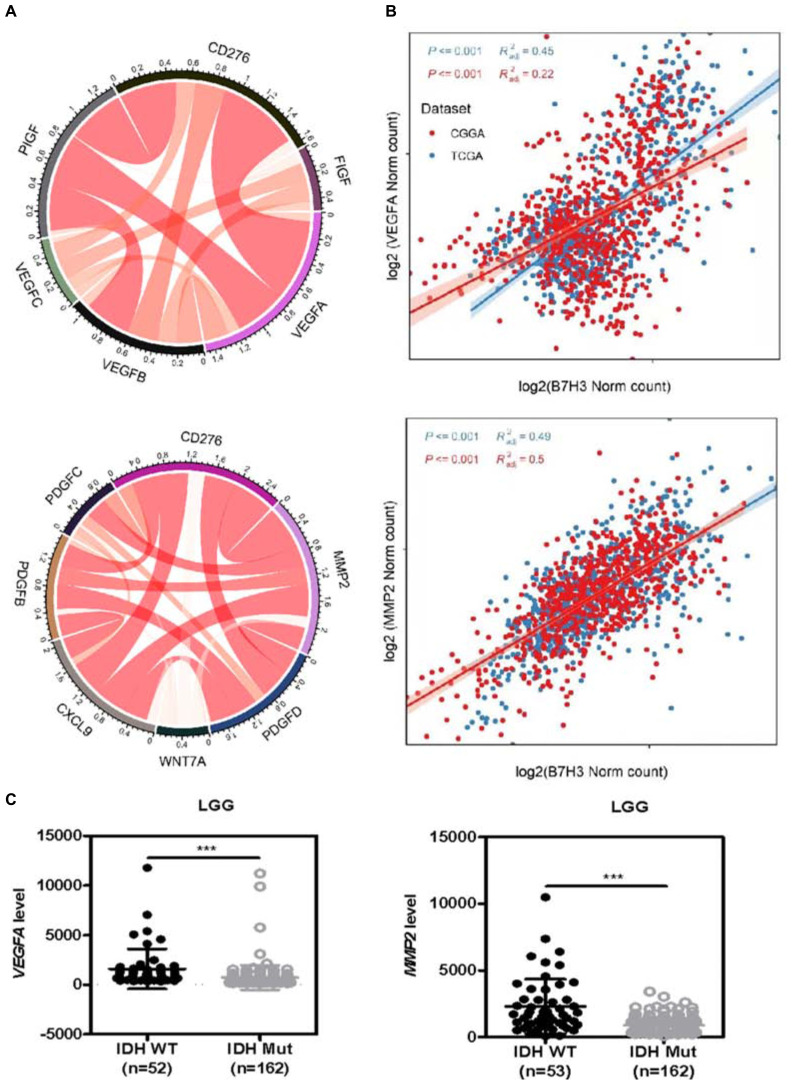
*B7H3* is positively correlated with *VEGFA* and *MMP2* in gliomas. **(A)** Circos analysis was performed to analyze the interactions among B7H3 and 11 key angiogenesis-related cytokines. **(B)** The relationship of *B7H3* and *VEGFA* or *MMP2* was analyzed according to the TCGA and CGGA databases. **(C)** Angiogenesis-related *VEGFA* and *MMP2* mRNA levels are decreased in IDH-mutated LGG according to the TCGA dataset. For *VEGFA* analysis, *n* = 52 in IDH-WT and *n* = 162 in IDH-MUT LGG, respectively, for *MMP2* analysis, *n* = 53 in IDH-WT and *n* = 162 in IDH-MUT LGG, respectively. ***denotes the *P* < 0.001 for the indicated comparisons.

Previous studies have reported that B7H3 is involved in angiogenesis in pancreatic carcinoma cells, colorectal cancer, and gliomas (Xie et al., 2016; Seaman et al., 2017; Wang et al., 2020). Considering that multiple growth factors and cytokines are involved in tumor angiogenesis (De Palma et al., 2017; Wang et al., 2020), we analyzed the interaction among B7H3 and 17 key angiogenesis-related growth factors and cytokines, including VEGFA, VEGFB, VEGFC, MMP2, PDGFA, PDGFB, PDGFC, PDGFD, FIGF, PIGF, PGF, FGF2, CXCL9, WNT7A, WNT7B, PECAM1, and THBS1 in gliomas by Circos and found that *B7H3* was significant positively correlated with *VEGFA* and *MMP2* as compared with the correlation between *B7H3* and other angiogenesis-related factors (Figure 4A and Supplementary Figure 5). Furthermore, the TCGA and CGGA database analysis showed that *VEGFA* (*R*^2^ = 0.45, and 0.22, respectively) and *MMP2* (*R*^2^ = 0.49, and 0.5, respectively) were tightly associated with *B7H3* in gliomas (Figure 4B), re-affirming the positive correlation between *B7H3* and *VEGFA* and *MMP2*. In addition, we found that the mRNA expressions of *VEGFA* and *MMP2* were both significantly decreased in IDH-mutated LGG compared to that in IDH-WT LGG by analyzing the TCGA database (Figure 4C), as was at a similar pattern of *B7H3* analyzed in Figure 1A. These results suggest that *B7H3* is positively correlated with *VEGFA* and *MMP2* in gliomas.

### Downregulation of B7H3 Is Associated With Reduced VEGFA Expression in IDH-Mutated Gliomas

**FIGURE 5:**
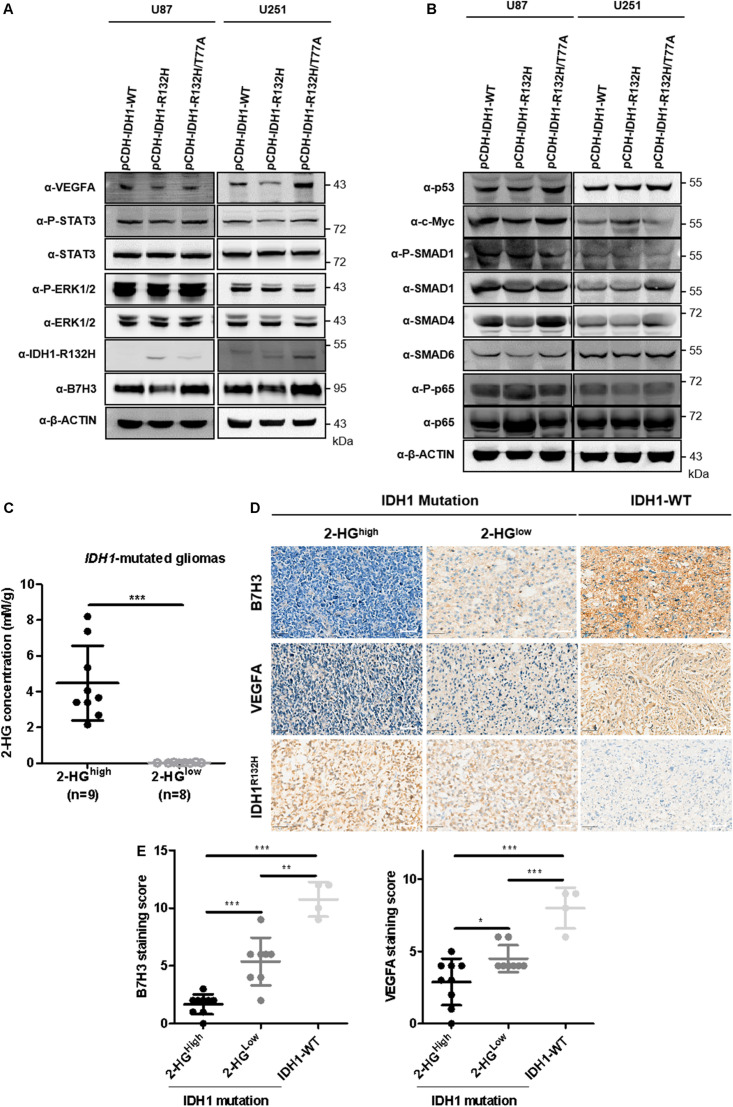
B7H3 downregulation is associated with reduced expression of VEGFA in IDH-mutated gliomas. **(A,B)** VEGFA is reduced accompanied by decreased B7H3 expression in IDH1-R132H U87 and U251 cells. Stably expressed IDH1-WT, IDH1-R132H, and IDH1-R132H/T77A U87 and U251 cells were collected and immunoblotting was performed with the indicated antibodies. All experiments were performed in triplicate and representative results were shown. **(C)** Absolute 2-HG quantification in IDH1-mutated gliomas. *N* = 9 in 2-HG^high^ and *n* = 8 in 2-HG^low^ *IDH1*-mutated gliomas, respectively. ***denotes the *P* < 0.001 for the indicated comparison. **(D,E)** B7H3 is positively associated with VEGFA in gliomas. IDH1-WT (*n* = 4), 2-HG^low^ IDH1-mutated gliomas (*n* = 8), and 2-HG^high^ IDH1-mutated gliomas (*n* = 9) were used for IHC staining with the indicated antibodies **(D)**, scale bar = 50 μM. Quantifications of B7H3 and VEGFA staining scores were shown in **(E)**. *denotes the *P* < 0.05, **denotes the *P* < 0.01, and ***denotes the *P* < 0.001 for the indicated comparisons.

Recent studies have shown that B7H3 promoted angiogenesis via inducing the expression of VEGFA in both pancreatic and colorectal cancers (Xie et al., 2016; Wang et al., 2020). Our earlier observation that *B7H3* has a positive correlation with *VEGFA* inspired us to examine the expression of VEGFA in IDH1-R132H glioma cells and we found that VEGFA expression was significantly decreased accompanied by reduced B7H3 in IDH1-R132H U87 cells (Figure 5A), while was increased to the comparable level in IDH1-R132H/T77A cells compared with IDH1-WT U87 cells (Figure 5A). Similarly, the consistent results of VEGFA and B7H3 levels was also observed in stable IDH1-WT, IDH1-R132H, and IDH1-R132H/T77A U251 cells (Figure 5A). These findings together show that VEGFA and B7H3 are both reduced in IDH1-R132H U87 and U251 cells. Multiple signaling pathways, such as STAT3 (Lei et al., 2018), ERK (Wang et al., 2019), p53 (Pal et al., 2001), c-Myc (Knies-Bamforth et al., 2004), TGF-β (Geng et al., 2013), and NF-kappaB (p65) (Wang et al., 2020) have been reported to be involved in regulating VEGFA expression. In addition, previous studies indicated that STAT3 (Li et al., 2017; Shi et al., 2019), ERK (Li et al., 2017), p53 (Son et al., 2019), c-Myc (Zhang et al., 2019), TGF-β (Zhang et al., 2019), and NF-kappaB (p65) (Xie et al., 2016) signaling pathways are the downstream targets of B7H3 in cancers. Hence, we hypothesized that one or more of these signaling pathways could change. To test our hypothesis, we examined the activities of the abovementioned six signaling pathways (by determining the levels of P-STAT3, P-ERK1/2, p53,c-Myc, P-SMAD, and P-p65) in IDH1-WT, IDH1-R132H, and IDH1-R132H/T77A U87 and U251 cells by immunoblotting. As shown in Figures 5A,B, the results revealed that IDH1-R132H significantly reduced the phosphorylation level of STAT3, while IDH1-R132H/T77A recovered P-STAT3 to a comparable level compared to IDH1-WT U87 cells. Likewise, similar results of the change of P-STAT3 were observed in IDH1-WT, IDH1-R132H, and IDH1-R132H/T77A U251 cells (Figures 5A,B). While the other five pathways, like ERK1/2, p53, c-MYC, TGF-β, and NF-kappaB (p65) were almost unchanged in IDH1-WT, IDH1-R132H, and IDH1-R132H/T77A U87 and U251 cells (Figures 5A,B).

In addition, we have previously demonstrated that the heterogenous 2-HG levels in 58 cases of IDH1-R132H mutated gliomas spanned from values close to 0–8.2 mM/g (Xu et al., 2019). To further detecting B7H3 and VEGFA expression levels in human glioma specimens, we randomly picked out 9 of 2-HG^high^, 8 of 2-HG^low^, and 4 IDH1-WT glioma samples (Figure 5C) to conduct IHC staining for B7H3 and VEGFA (Figure 5D). In agreement with our earlier observations, B7H3 and VEGFA were significantly decreased in IDH1-mutated gliomas compared to that in IDH1-WT glioma samples (Figures 5D,E). Surprisingly, a further drop of B7H3 and VEGFA was observed in 2-HG^high^ gliomas compared to that in 2-HG^low^ glioma sections (Figures 5D,E).

Taken together, these findings support a notion that downregulation of B7H3 is associated with reduced VEGFA in IDH-mutated gliomas, and B7H3 and VEGFA further decrease in IDH-mutated gliomas with highly accumulated 2-HG level.

## Discussion

In spite of recent findings and new insights into the genomic changes associated with gliomas, the diagnosis, treatment and prognosis all got improved. Recently, B7 family members have attracted more attention, especially following the successful treatment in advanced melanoma, lung cancer, and other solid tumors with anti-PD1/PDL1 and anti-CTLA4 therapies (Sznol and Chen, 2013; Wolchok et al., 2013). Among the B7 family proteins, B7H3 is the most highly expressed in GBM (Zhang et al., 2019). However, the expression preference of B7H3 in different subtypes of gliomas is largely unknown. In this study, we find that accumulation of D-2-HG by IDH mutation significantly reduces the protein level of B7H3 in gliomas. A potential mechanism is that IDH mutation accelerates the autophagy flux, thus promoting B7H3 degradation through autophagy. Our bioinformatics analysis, immunoblotting, and IHC staining reveal that B7H3 is positively associated with VEGFA in gliomas. B7H3 and VEGFA are decreased in IDH-mutated gliomas and these two proteins are further reduced when D-2-HG accumulates highly in IDH-mutated gliomas.

The mRNA level of *B7H3* was lower in IDH-mutated gliomas compared to IDH-WT gliomas according to the TCGA and CGGA databases (Wang et al., 2018; Zhang et al., 2018), which was validated again in our study. However, the protein level of B7H3 in IDH-mutated and WT gliomas is unknown yet. We find here that IDH mutation significantly reduces the expression of B7H3 protein, and it is D-2-HG produced by mutant IDH that is responsible for the downregulated B7H3 protein. In addition, we further find that B7H3 is a stable protein, and it can be most likely degraded through autophagy pathway.

Tumor angiogenesis is a hallmark of cancers, and abnormal angiogenesis network is now a big challenge for clinical therapy. Antibodies targeting VEGF-related signaling has become a popular and promising therapy for cancers (Rivera and Bergers, 2015). In this study, we found that B7H3 was positively correlated with VEGFA in gliomas. B7H3 and VEGFA were both downregulated in IDH-mutated glioma cells and tissue specimens. Notably, the expression of B7H3 and VEGFA were both further reduced in 2-HG^high^ gliomas compared to that in 2-HG^low^ gliomas. However, further investigation is needed to explore whether and how B7H3 regulates VEGFA in gliomas.

In summary, we show in this study that B7H3 is significantly downregulated in IDH-mutated gliomas due to 2-HG accumulation, which is through the autophagy degradation pathway. The protein level of B7H3 is positively correlated with VEGFA, and both proteins are reduced in *IDH1*-mutated glioma samples and further decreased in 2-HG^high^ gliomas. Our study demonstrates that B7H3 is preferentially overexpressed in *IDH-*WT gliomas and could serve as a potential target for future precise glioma treatment. This also reminds us that the future target therapy might need molecular guide.

## Data Availability Statement

The datasets presented in this study can be found in online repositories. The names of the repository/repositories and accession number(s) can be found below: EBI MetaboLights, accession no: MTBLS2692.

## Ethics Statement

This study has been granted ethics approval from the Huashan hospital Ethics Committee. Consent forms were obtained from all patients after approval by local ethics committee.

## Author Contributions

WH, YM, HY, DY, and MZ conceived the general framework of this study. MZ designed and performed experiments. HZ and MZ performed clinical data collection and analysis. HZ, MF, and JgZ performed bioinformatics analysis. CZ performed LC-MS/MS assay. YL, FF, JsZ, and HX provided other experimental materials and equipment. MZ, WH, and HY prepared the manuscript. All authors read and approved the final manuscript.

## Conflict of Interest

The authors declare that the research was conducted in the absence of any commercial or financial relationships that could be construed as a potential conflict of interest.

## Footnotes

1 https://www.uniprot.org/

2 http://www.jalview.org/

3 https://www.cancer.gov/

4 http://www.cgga.org.cn/

5 https://www.r-project.org/
