# Supplementary material for: The Inhibition of B7H3 by 2-HG Accumulation Is Associated With Downregulation of VEGFA in IDH Mutated Gliomas
Source: Front Cell Dev Biol. 2021 May 17;9:670145. doi: 10.3389/fcell.2021.670145 (PMC8165280; doi:10.3389/fcell.2021.670145)
Supplement: Supplementary file 1 [file Data_Sheet_1.pdf]

Supplementary Material

Supplementary Figure 1, related to Figure 2.

Cell-permeable D-2-HG treatment accumulates 2-HG in U87 and U251 cells

Supplementary Figure 2, related to Figure 2.

Stable overexpression of IDH1-R132H accumulates 2-HG in U87 and U251 cell lines

Supplementary Figure 3, related to Figure 3.

MG132, BTZ, and MLN4924 cannot accumulate B7H3 protein level in IDH1-R132H U87 cells

Supplementary Figure 4, related to Figure 3.

Leupeptin and Baf-A1 accumulate B7H3 protein level in IDH1-R132H U87 cells

Supplementary Figure 5, related to Figure 4.

Correlation between *B7H3* and angiogenesis-related cytokines in gliomas

Supplementary Figure 1. Cell-permeable D-2-HG treatment accumulates 2-HG in U87 and U251 cells.

(A) Expression of B7H3 in glioma cell lines. The protein level of B7H3 was detected in U87, U251, A172 and U118 glioma cells with the indicated antibodies. HA (Human astrocyte) was as a normal control cell line.

(B) Verification of cell-permeable D-2-HG in U87 and U251 cells by LC-MS/MS. U87 and U251 cells were treated with cell-permeable octyl-D-2HG, octyl-L-2HG, or DMSO. After cultured for 24 hours, the cells were collected and subjected to LC-MS/MS for 2-HG detection as described in 'MATERIALS AND METHODS'.

All experiments were performed in triplicate and representative results were shown.

Supplementary Figure 2. Stable overexpression of IDH1-R132H accumulates 2-HG in U87 and U251 cell lines.

(A) Verification of 2-HG in stable IDH1-R132H U87 cells by LC-MS/MS. 2×10^5 cells were seeded in the six-well plates. After 24 hours, the cells were subjected to LC-MS/MS for 2-HG detection as described in 'MATERIALS AND METHODS'. N = 3. Relative level of 2-HG was quantified (Left) and representative MS result was presented (Right).

(B) Verification of 2-HG in stable IDH1-R132H U251 cells by LC-MS/MS. Stable U251 cells were seeded and subjected to LC-MS/MS for 2-HG detection as described in (A). N = 3. Relative level of 2-HG was quantified (Left) and representative MS result was presented (Right).

(C) Verification of stably ectopic IDH1 expression in U251 cells. Cells were collected for immunoblotting with the indicated antibodies. All experiments were performed in triplicate and representative results were shown.

Supplementary Figure 3. MG132, BTZ, and MLN4924 cannot accumulate B7H3 protein level in IDH1-R132H U87 cells.

(A-C) Cells were treated with MG132, BTZ, or MLN4924 for the indicated concentrations and time. After cell collection, immunoblotting was performed with the indicated antibodies. All experiments were performed in triplicate and representative results were shown.

Supplementary Figure 4. Leupeptin and Baf-A1 accumulate B7H3 protein level in IDH1-R132H U87 cells.

(A-E) Inhibition of autophagy by leupeptin and Baf-A1 blocks the degradation of B7H3 in stable IDH1-R132H U87 cells. Cells were treated with MG132, BTZ, MLN4924, leupeptin, or Baf-A1 for

the indicated concentrations and time points. Immunoblotting was performed with the indicated antibodies. All experiments were performed in triplicate and representative results were shown.

Supplementary Figure 5. Correlation between *B7H3* and angiogenesis-related cytokines in gliomas.

Circos analysis was performed to analyze the interactions among *B7H3* and 6 key angiogenesis-related cytokines.
