# Supplementary figures and images for: The Inhibition of B7H3 by 2-HG Accumulation Is Associated With Downregulation of VEGFA in IDH Mutated Gliomas
Source: Front Cell Dev Biol. 2021 May 17;9:670145. doi: 10.3389/fcell.2021.670145 (PMC8165280; doi:10.3389/fcell.2021.670145)

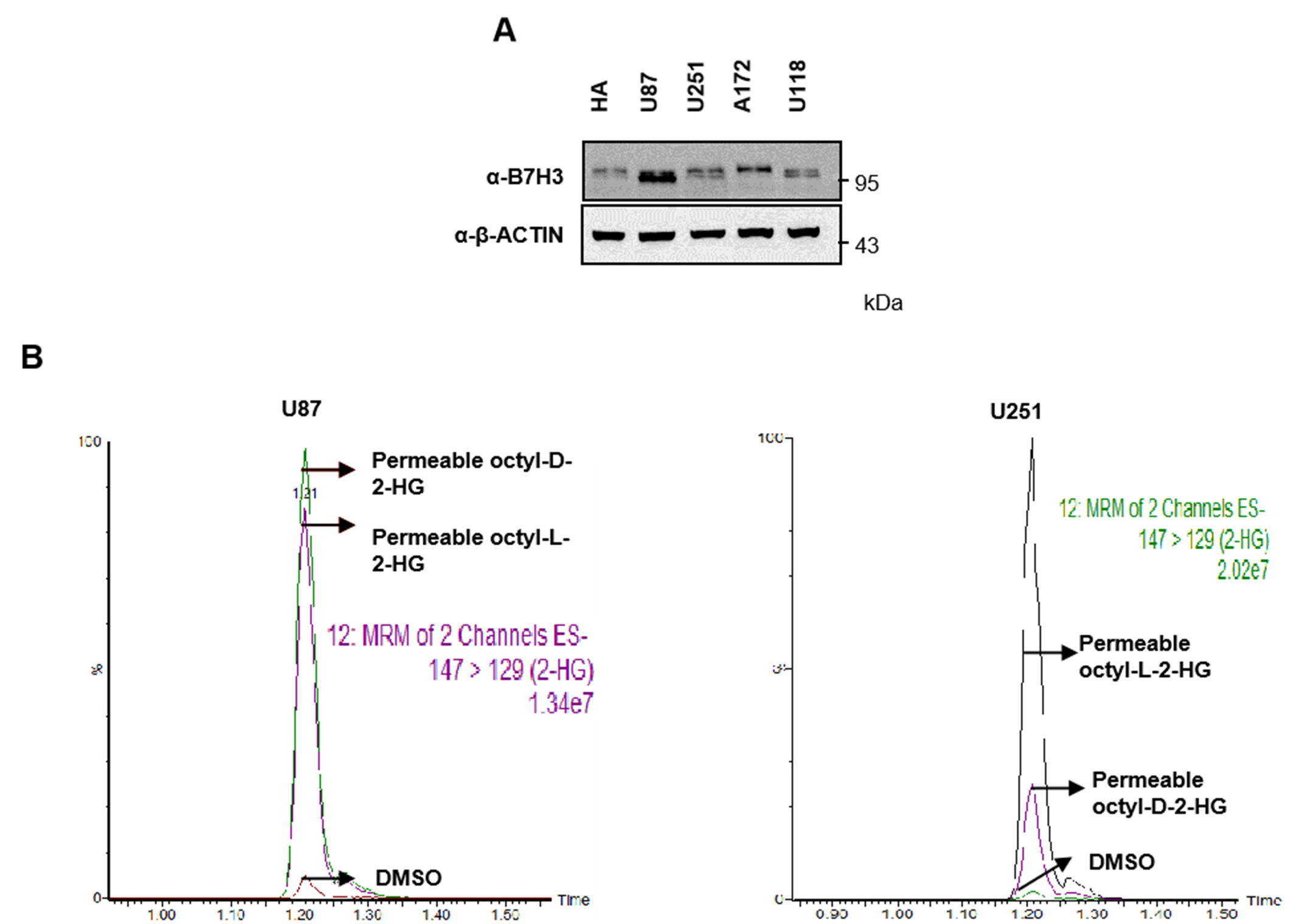

Supplement: Supplementary file 2 [file Data_Sheet_2.zip › Supplementary Figures-300dpi/Supplementary Figure 1.tif]

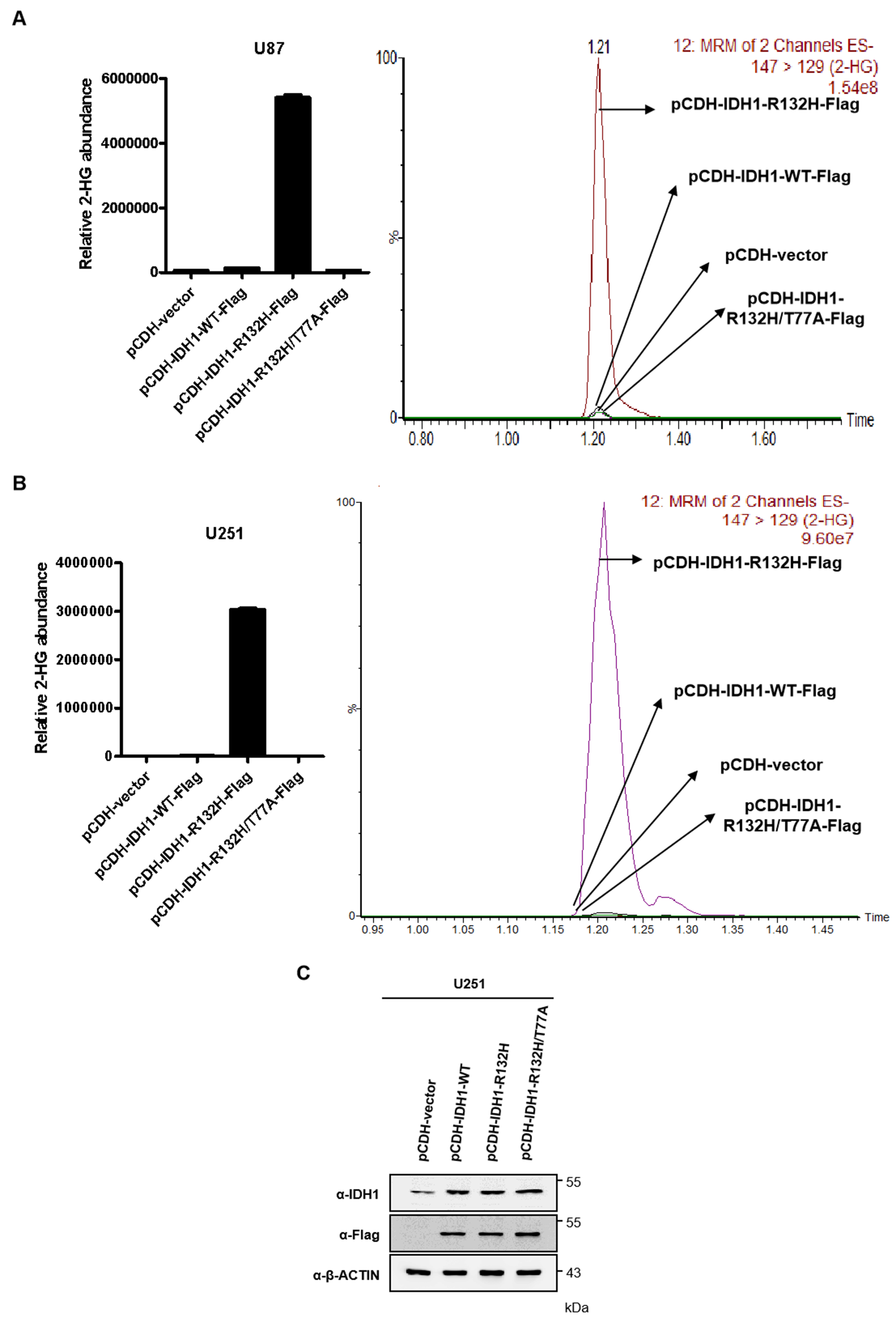

Supplement: Supplementary file 2 [file Data_Sheet_2.zip › Supplementary Figures-300dpi/Supplementary Figure 2.tif]

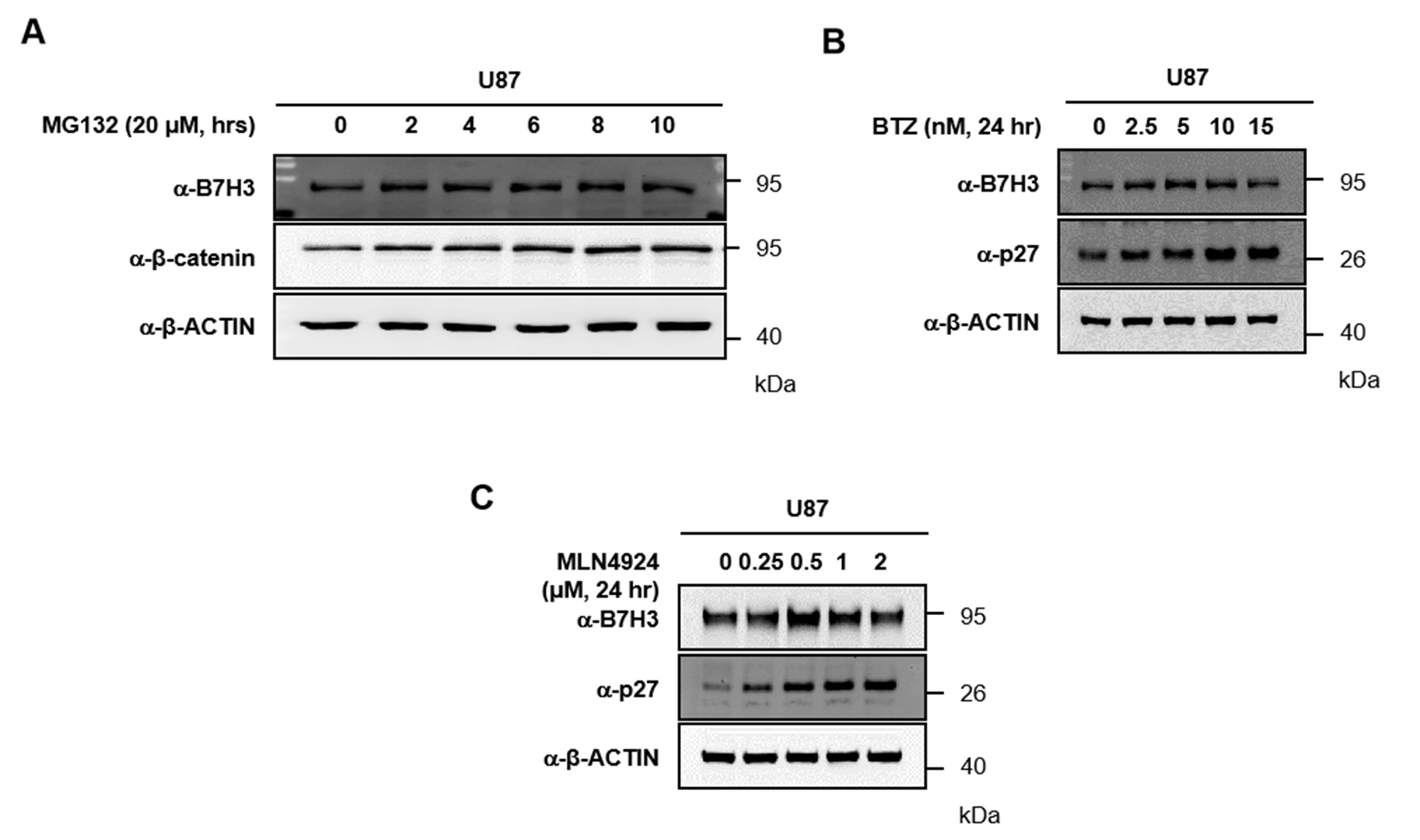

Supplement: Supplementary file 2 [file Data_Sheet_2.zip › Supplementary Figures-300dpi/Supplementary Figure 3.tif]

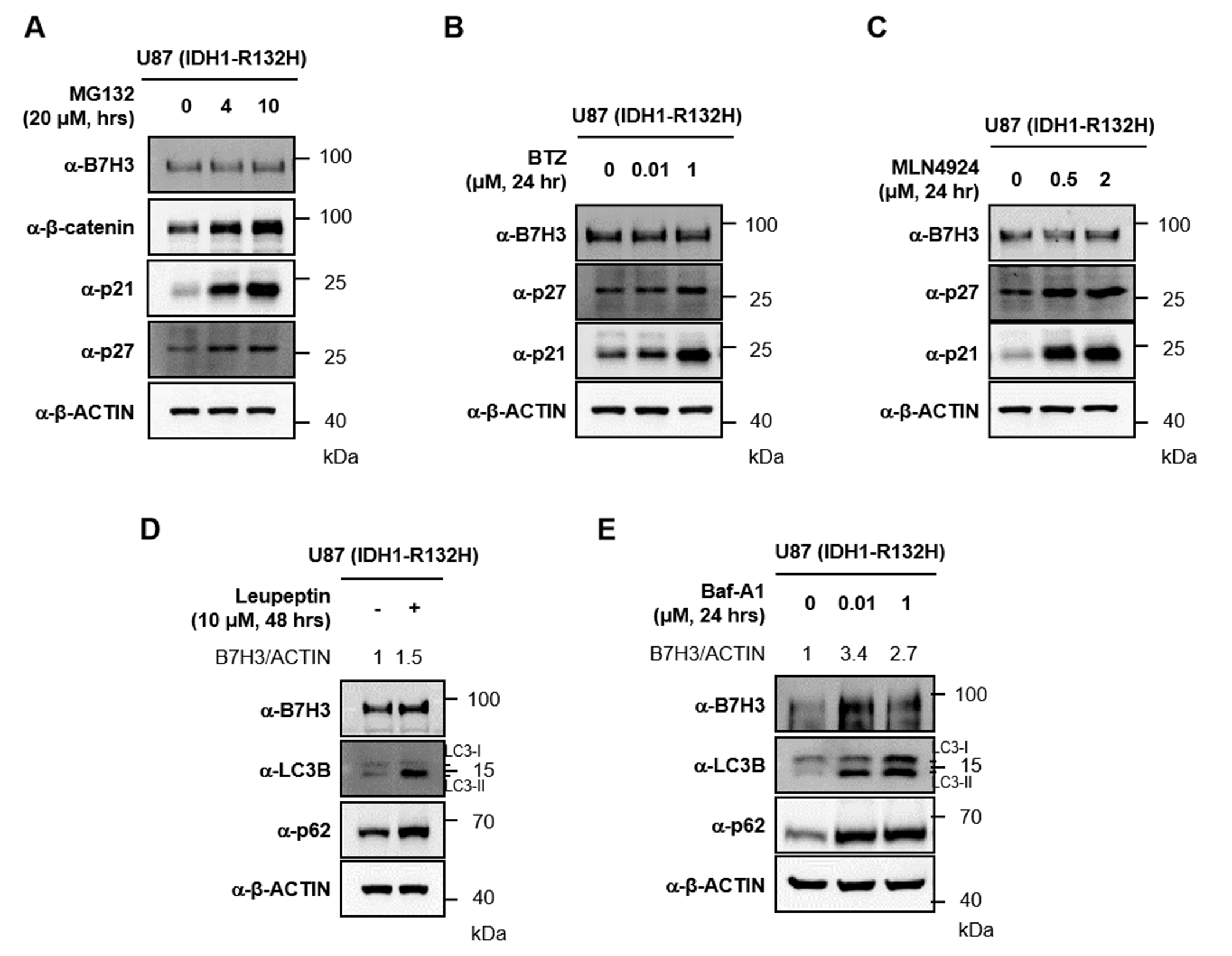

Supplement: Supplementary file 2 [file Data_Sheet_2.zip › Supplementary Figures-300dpi/Supplementary Figure 4.tif]

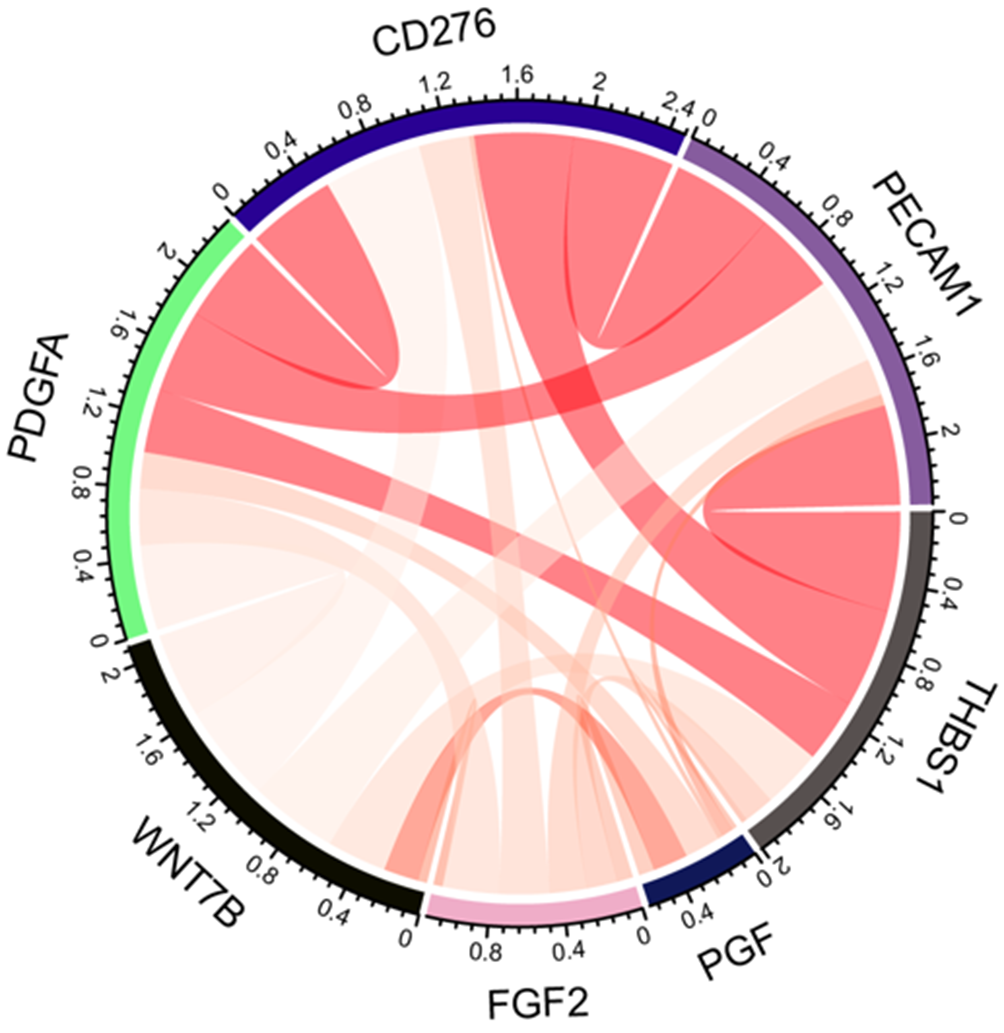

Supplement: Supplementary file 2 [file Data_Sheet_2.zip › Supplementary Figures-300dpi/Supplementary Figure 5.tif]
