# Supplementary material for: The Inhibition of B7H3 by 2-HG Accumulation Is Associated With Downregulation of VEGFA in IDH Mutated Gliomas
Source: Front Cell Dev Biol. 2021 May 17;9:670145. doi: 10.3389/fcell.2021.670145 (PMC8165280; doi:10.3389/fcell.2021.670145)
Supplement: Supplementary file 3 [file Data_Sheet_3.zip › Original data/Original data for Graphs-B7H3.pdf]

												T-TEST
Figure 1B	IDH1-WT	6.713102	1	0.935045	6.810178	2.182495	2.792218	1.573214	1.181619	4.90036	2.563979	7.60244	
	IDH1-R132H	1.767073	1.680093	2.289038	0.416181	2.316632	1.17704	0.874846	0.398735	1.252627	0.971372	0.650674	0.011250945

Figure 2E	Vector	IDH1-WT	IDH1-R132H	IDH1-R132H/T77A
	0	0	0.02441906	0
	0	0	0.02524431	0
	0	0	0.02555516	0

Figure 2F	Vector	IDH1-WT	IDH1-R132H	IDH1-R132H/T77A
	0	0	0.03040269	0
	0	0	0.03120317	0
	0	0	0.03002414	0

Figure 3A	Time (hrs)	DMSO	Octyl-D-2-HG
	0	1	1
	2	1.32	0.96
	4	1.22	0.90
	6	1.01	0.84
	8	0.96	0.84
	10	0.92	0.88

Figure 3B		IDH1-WT	IDH1-R132H
	0	1	1.00
	2	0.78	1.08
	4	0.88	0.83
	6	0.72	0.89
	8	1.19	0.93
	10	1.02	1.03

											T-TEST
Figure 5C	2-HG^{high}	2.151029	3.664559	8.199384	7.371282	5.347041	2.687876	3.405324	3.391752	4.065257	
	2-HG^{low}	0.008518069	0.01731743	0.06759579	0.05561548	0.01142598	0.005407252	0.02850761	0.02065625		2.36903E-05

											T-TEST
Figure 5E-Left	2-HG^{High}	2	1	2	2	3	2	1	2	0	0.001000724
	2-HG^{Low}	6	2	4	6	6	9	4	6		0.00017974
	IDH1-WT	12	9	10	12						2.28169E-08

											T-TEST
Figure 5E-Right	2-HG^{High}	2	1	4	3	4	5	3	4	0	0.000391287
	2-HG^{Low}	4	4	4	4	4	6	6	4		0.025671369
	IDH1-WT	9	6	8	9						0.000203878

Supplementary Figure 2A	pCDH-vector	71168.36	73184.85	73175.2	78797.34	72429.3	66992.8
	pCDH-IDH1-WT-Flag	136527.3	140127.8	150313.6	153369.8	146897.8	143956.6
	pCDH-IDH1-R132H-Flag	5651144	5593107	5320353	5438430	5341165	5093734
	pCDH-IDH1-R132H/T77A-Flag	73481.2	69661.24	81181.45	78447.94	72764.83	70108.58

Supplementary Figure 2B	pCDH-vector	5636.608	6038.706	5880.063	6794.702	8072.236	7590.518
	pCDH-IDH1-WT-Flag	24773.46	24767.68	23392.56	23985.55	24007.15	23972.2
	pCDH-IDH1-R132H-Flag	2987198	3122136	3021224	3088197	3042240	3013217
	pCDH-IDH1-R132H/T77A-Flag	6933.931	6329.045	6944.408	7128.895	6750.338	7046.955
