# Supplementary material for: The Inhibition of B7H3 by 2-HG Accumulation Is Associated With Downregulation of VEGFA in IDH Mutated Gliomas
Source: Front Cell Dev Biol. 2021 May 17;9:670145. doi: 10.3389/fcell.2021.670145 (PMC8165280; doi:10.3389/fcell.2021.670145)

Figure 1B

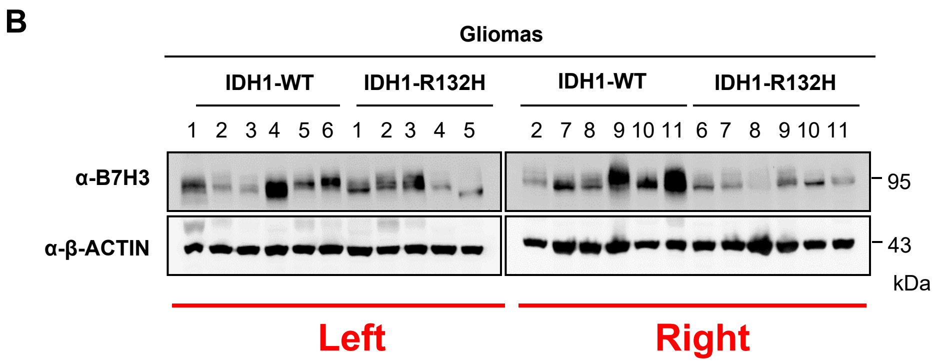
B
Gliomas
IDH1-WT
IDH1-R132H
IDH1-WT
IDH1-R132H
1 2 3 4 5 6 1 2 3 4 5
2 7 8 9 10 11 6 7 8 9 10 11
α-B7H3
α-β-ACTIN
95
43
kDa
Left
Right

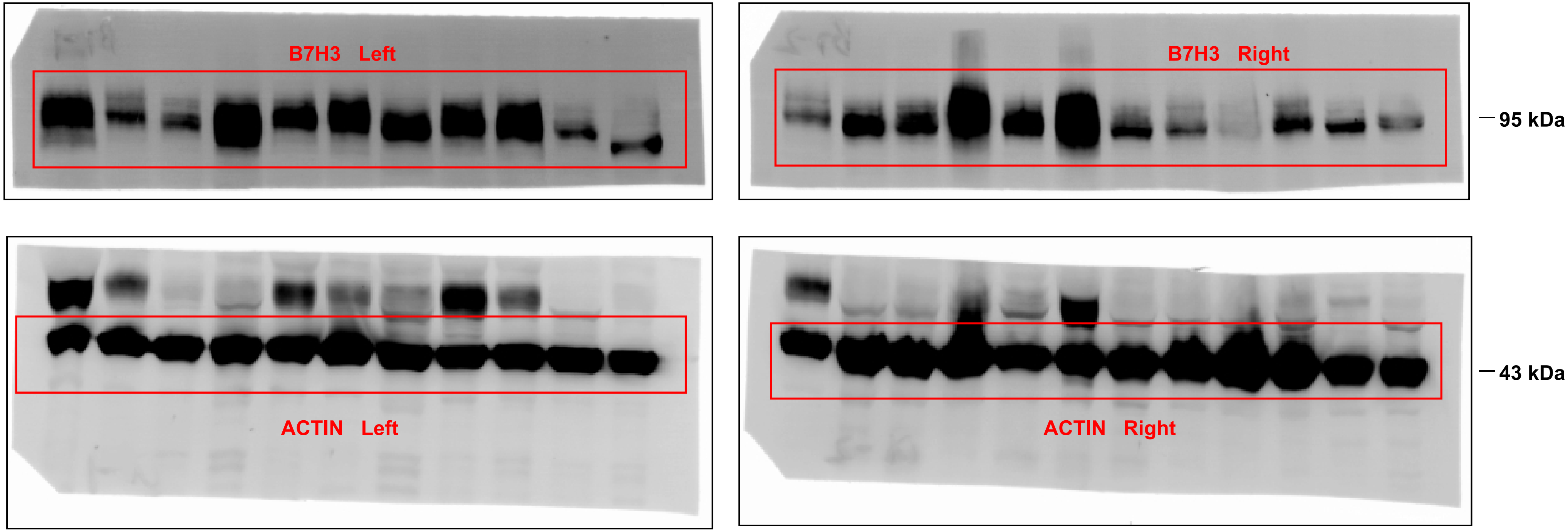
B7H3 Left
B7H3 Right
95 kDa
ACTIN Left
ACTIN Right
43 kDa

Figure 2A-B

A

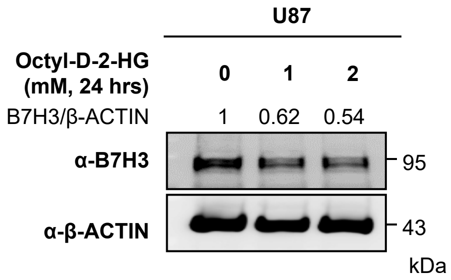

B

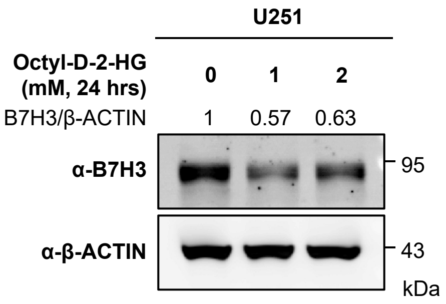

A

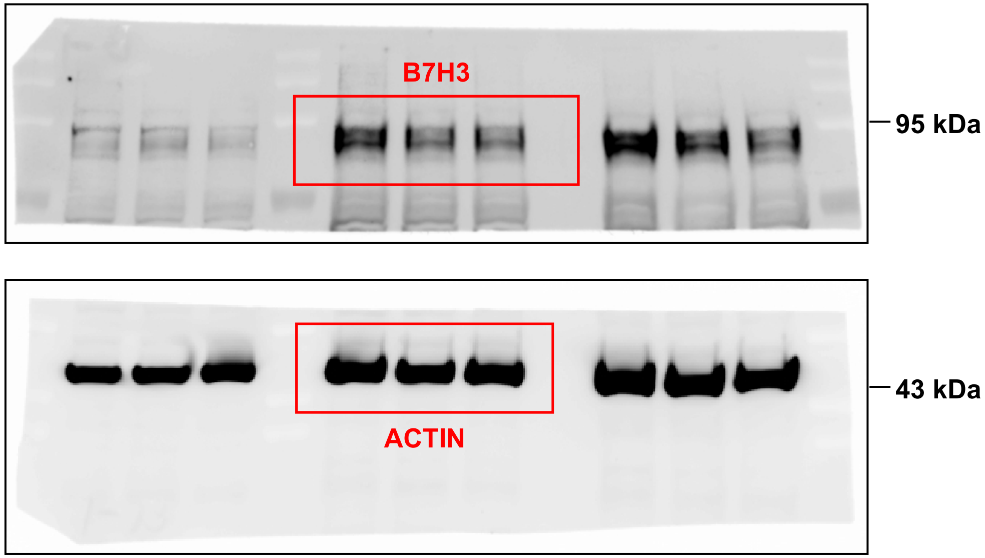

B

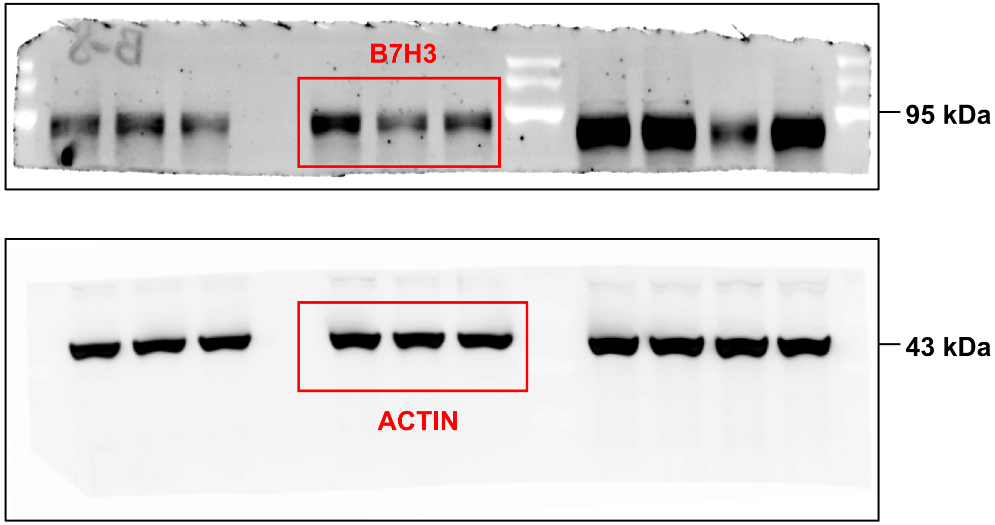

Figure 2C-D

C

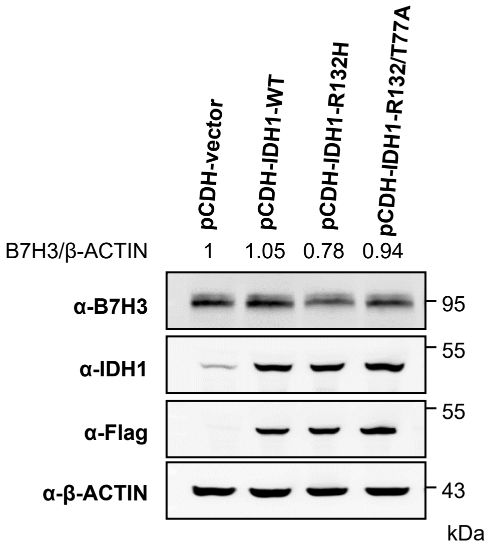

D

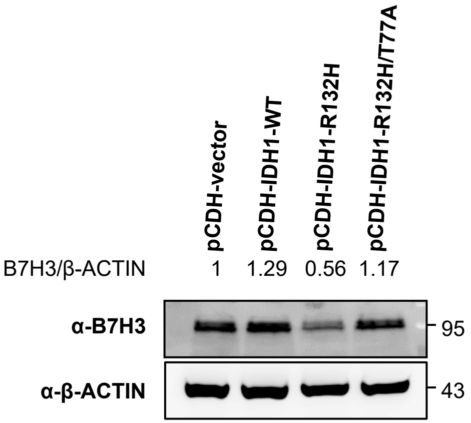

C

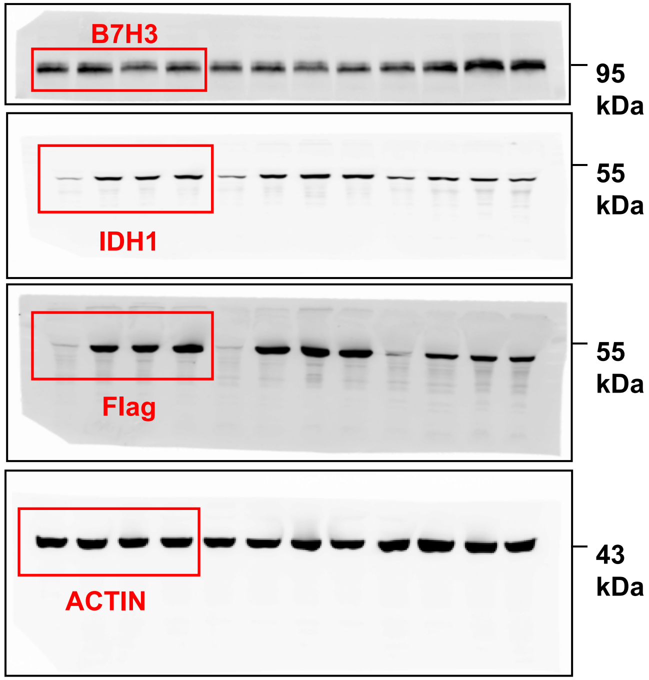

D

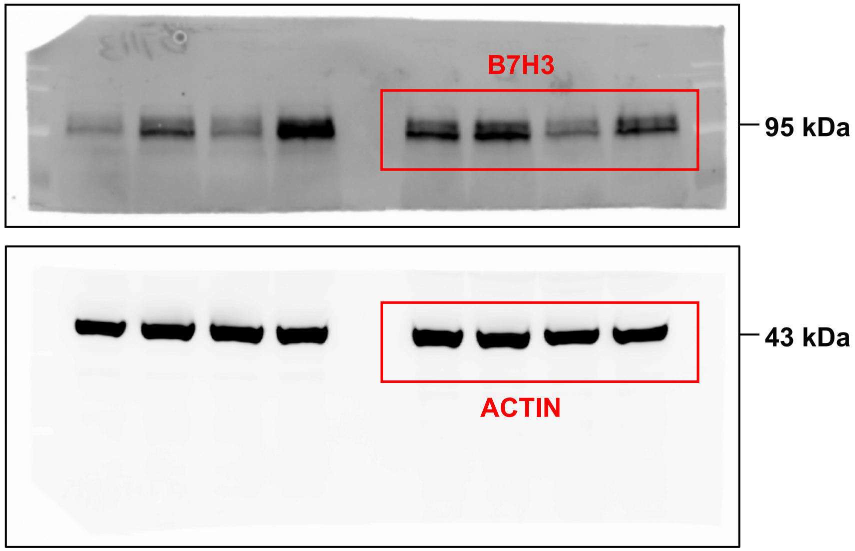

Figure 3A-B

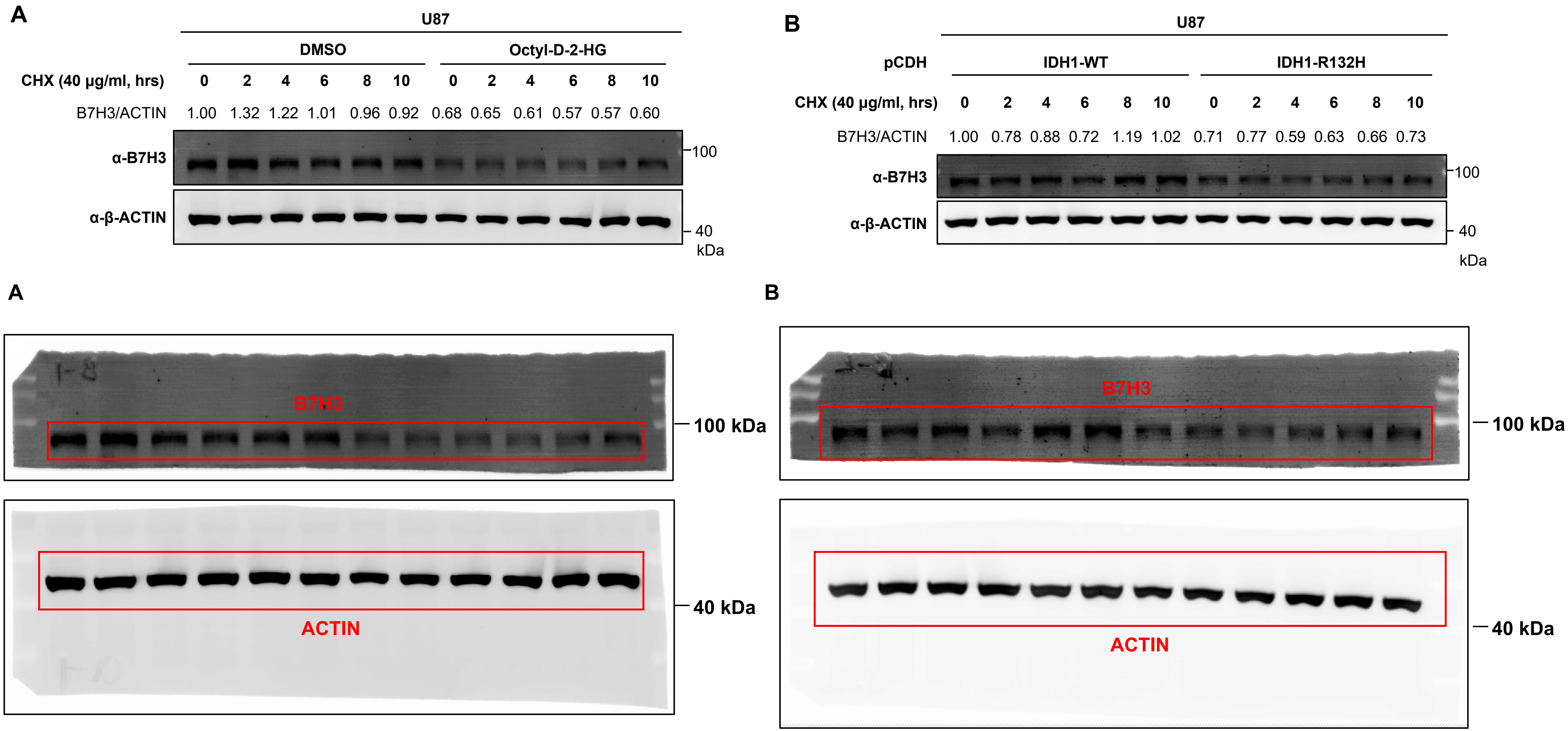
A
U87
DMSO
Octyl-D-2-HG
CHX (40 µg/ml, hrs)
0 2 4 6 8 10 0 2 4 6 8 10
B7H3/ACTIN
1.00 1.32 1.22 1.01 0.96 0.92 0.68 0.65 0.61 0.57 0.57 0.60
α-B7H3
α-β-ACTIN
100
40
kDa
B
U87
pCDH
IDH1-WT
IDH1-R132H
CHX (40 µg/ml, hrs)
0 2 4 6 8 10 0 2 4 6 8 10
B7H3/ACTIN
1.00 0.78 0.88 0.72 1.19 1.02 0.71 0.77 0.59 0.63 0.66 0.73
α-B7H3
α-β-ACTIN
100
40
kDa
A
B7H3
100 kDa
ACTIN
40 kDa
B
B7H3
100 kDa
ACTIN
40 kDa

Figure 3C-D

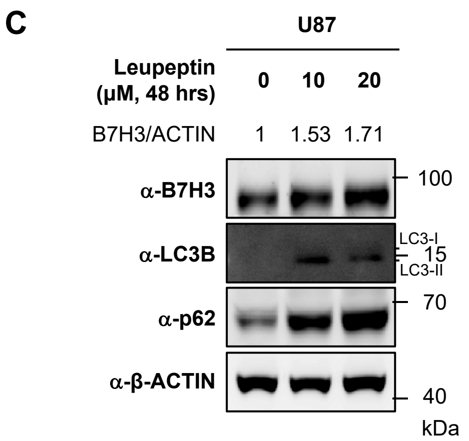
C
U87
Leupeptin (μM, 48 hrs)
0
10
20
B7H3/ACTIN
1
1.53
1.71
α-B7H3
α-LC3B
α-p62
α-β-ACTIN
100
LC3-I
15
LC3-II
70
40
kDa

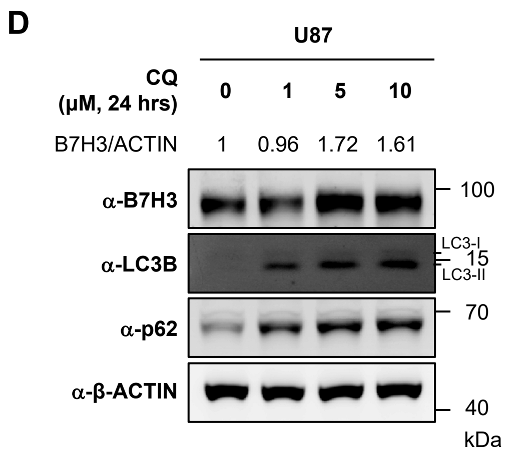
D
U87
CQ (μM, 24 hrs)
0
1
5
10
B7H3/ACTIN
1
0.96
1.72
1.61
α-B7H3
α-LC3B
α-p62
α-β-ACTIN
100
LC3-I
15
LC3-II
70
40
kDa

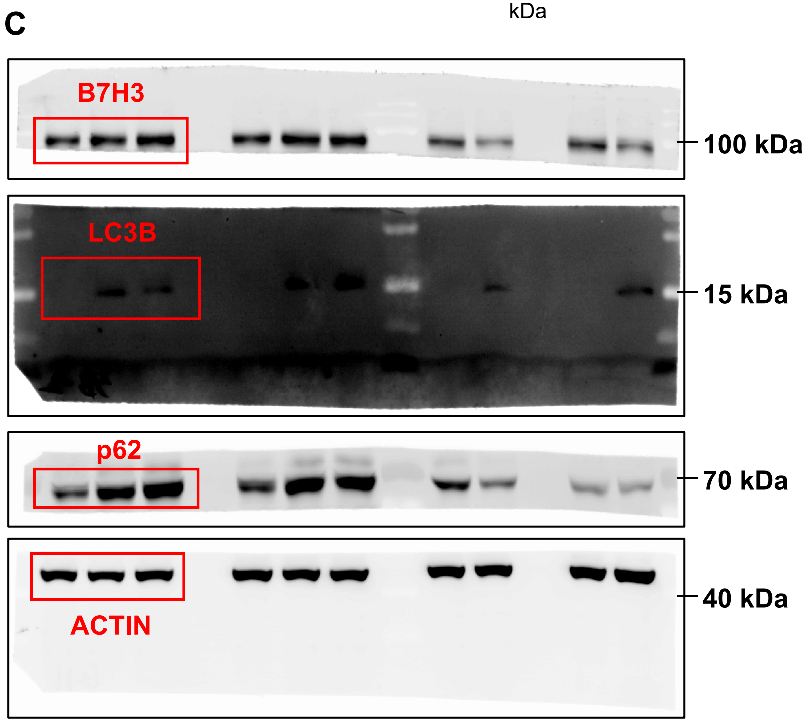
C
B7H3
100 kDa
LC3B
15 kDa
p62
70 kDa
ACTIN
40 kDa

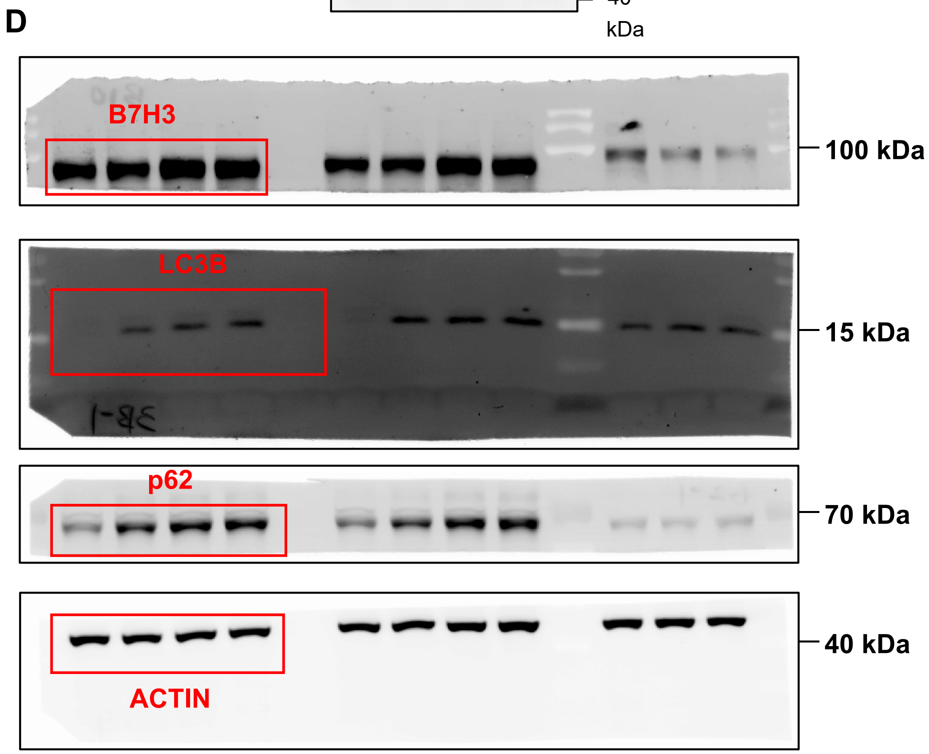
D
B7H3
100 kDa
LC3B
15 kDa
p62
70 kDa
ACTIN
40 kDa

Figure 3E-F

E

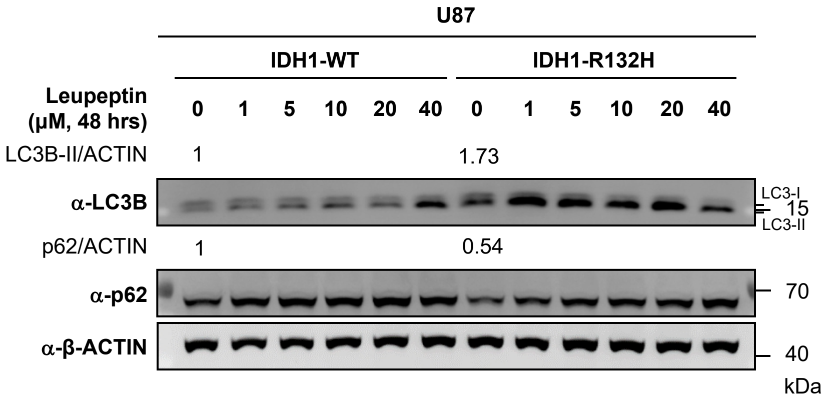

F

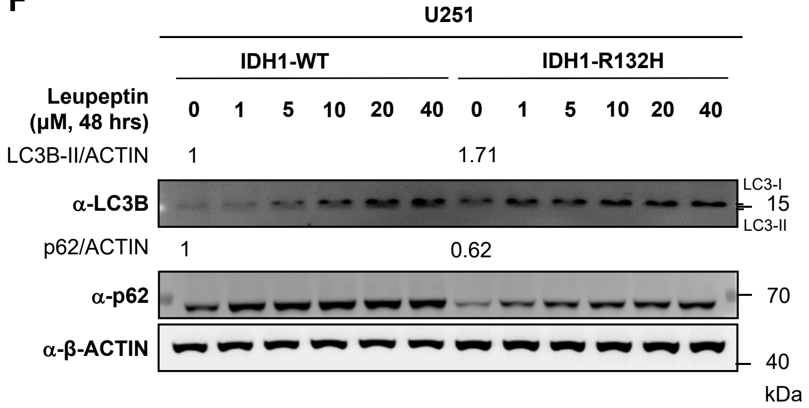

E

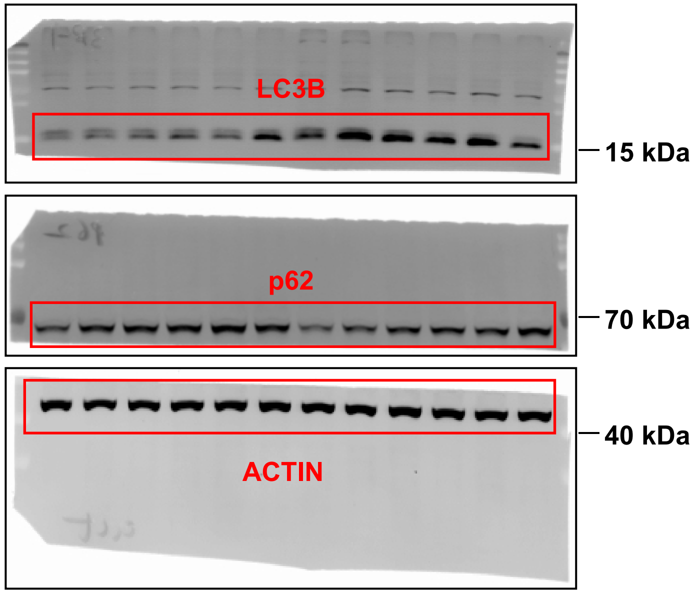

F

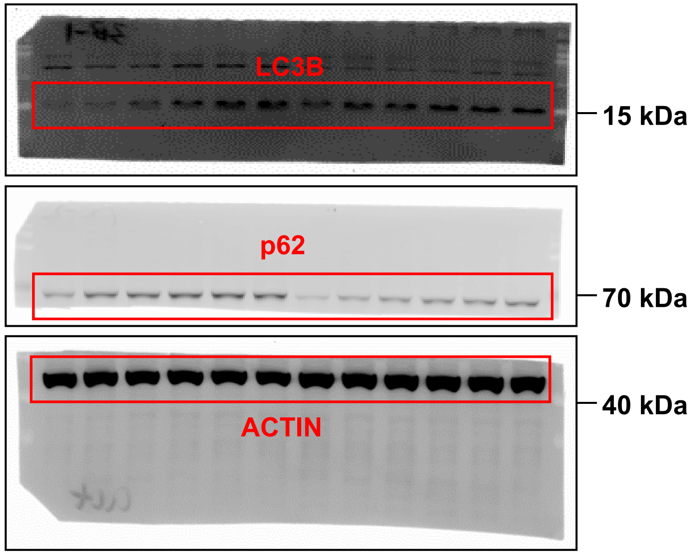

Figure 3H

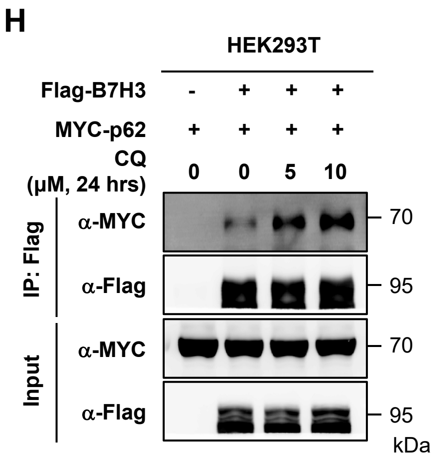
H
HEK293T
Flag-B7H3 - + + +
MYC-p62 + + + +
CQ (μM, 24 hrs) 0 0 5 10
IP: Flag
α-MYC
α-Flag
Input
α-MYC
α-Flag
70
95
70
95
kDa

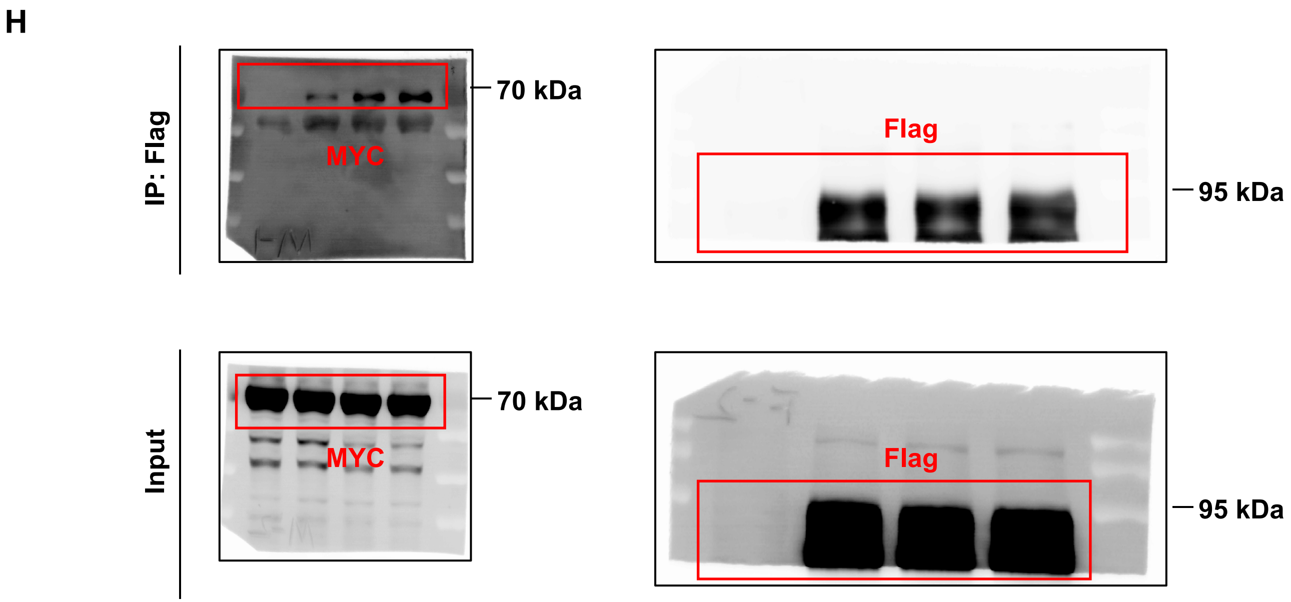
H
IP: Flag
MYC
70 kDa
Flag
95 kDa
Input
MYC
70 kDa
Flag
95 kDa

Figure 5A

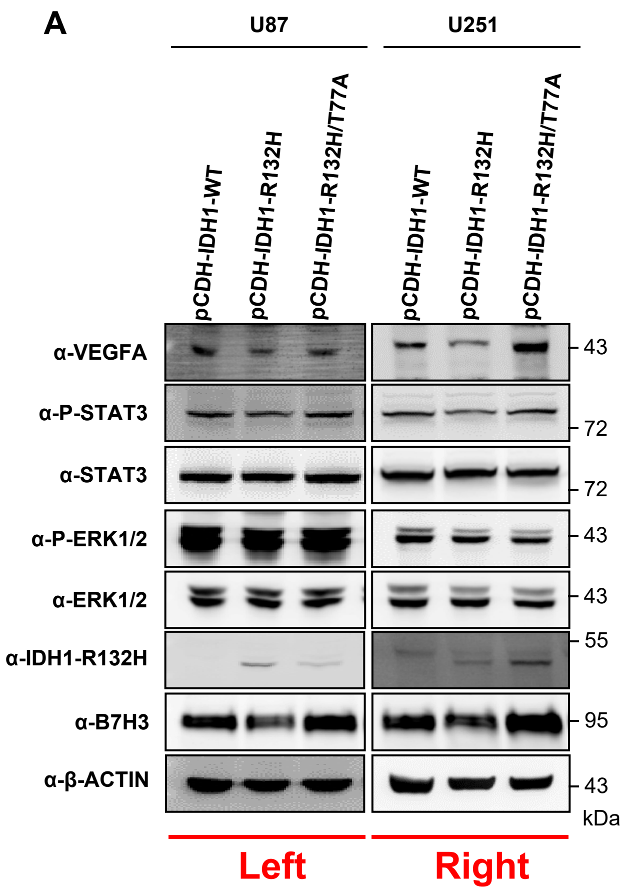
A
U87
U251
pCDH-IDH1-WT
pCDH-IDH1-R132H
pCDH-IDH1-R132H/T77A
pCDH-IDH1-WT
pCDH-IDH1-R132H
pCDH-IDH1-R132H/T77A
α-VEGFA
α-P-STAT3
α-STAT3
α-P-ERK1/2
α-ERK1/2
α-IDH1-R132H
α-B7H3
α-β-ACTIN
43
72
72
43
43
55
95
43
kDa
Left
Right

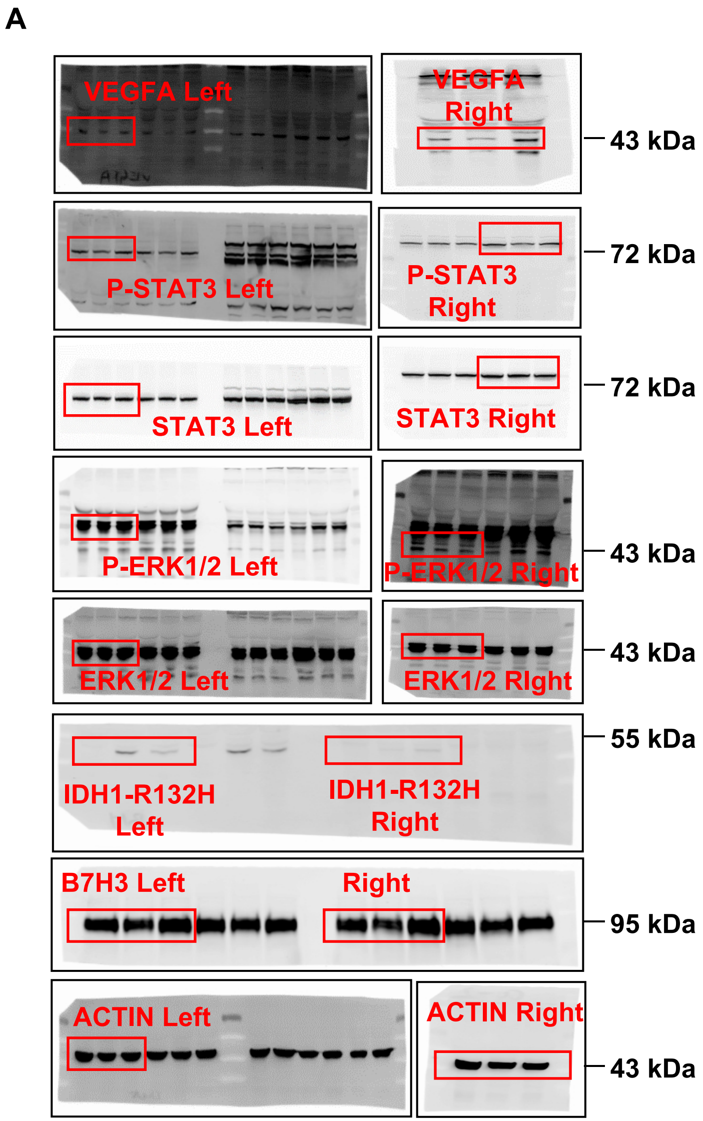
A
VEGFA Left
VEGFA Right
43 kDa
P-STAT3 Left
P-STAT3 Right
72 kDa
STAT3 Left
STAT3 Right
72 kDa
P-ERK1/2 Left
P-ERK1/2 Right
43 kDa
ERK1/2 Left
ERK1/2 RIght
43 kDa
IDH1-R132H Left
IDH1-R132H Right
55 kDa
B7H3 Left
Right
95 kDa
ACTIN Left
ACTIN Right
43 kDa

Figure 5B

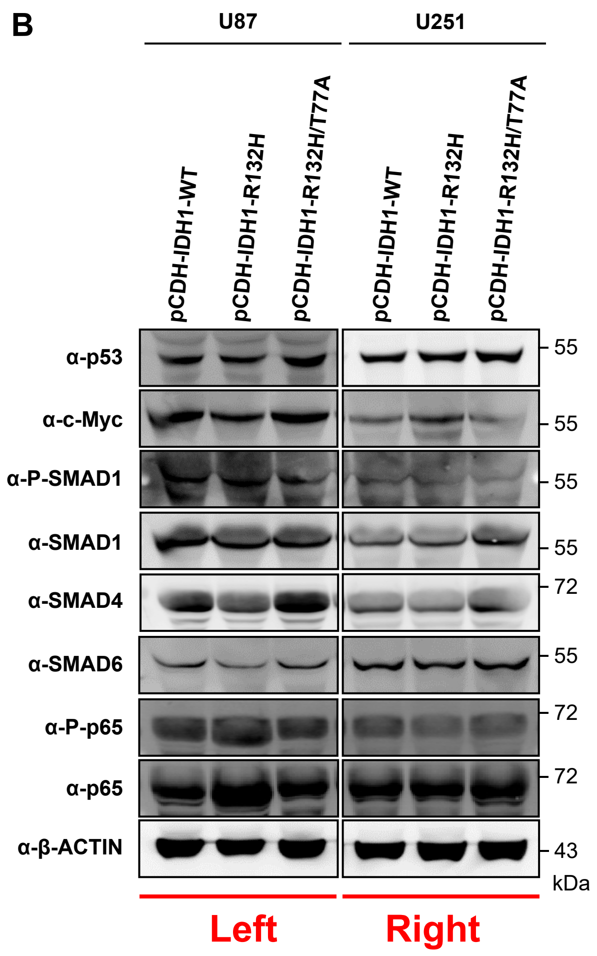
B
U87
U251
pCDH-IDH1-WT
pCDH-IDH1-R132H
pCDH-IDH1-R132H/T77A
pCDH-IDH1-WT
pCDH-IDH1-R132H
pCDH-IDH1-R132H/T77A
α-p53
α-c-Myc
α-P-SMAD1
α-SMAD1
α-SMAD4
α-SMAD6
α-P-p65
α-p65
α-β-ACTIN
55
55
55
55
72
55
72
72
43
kDa
Left
Right

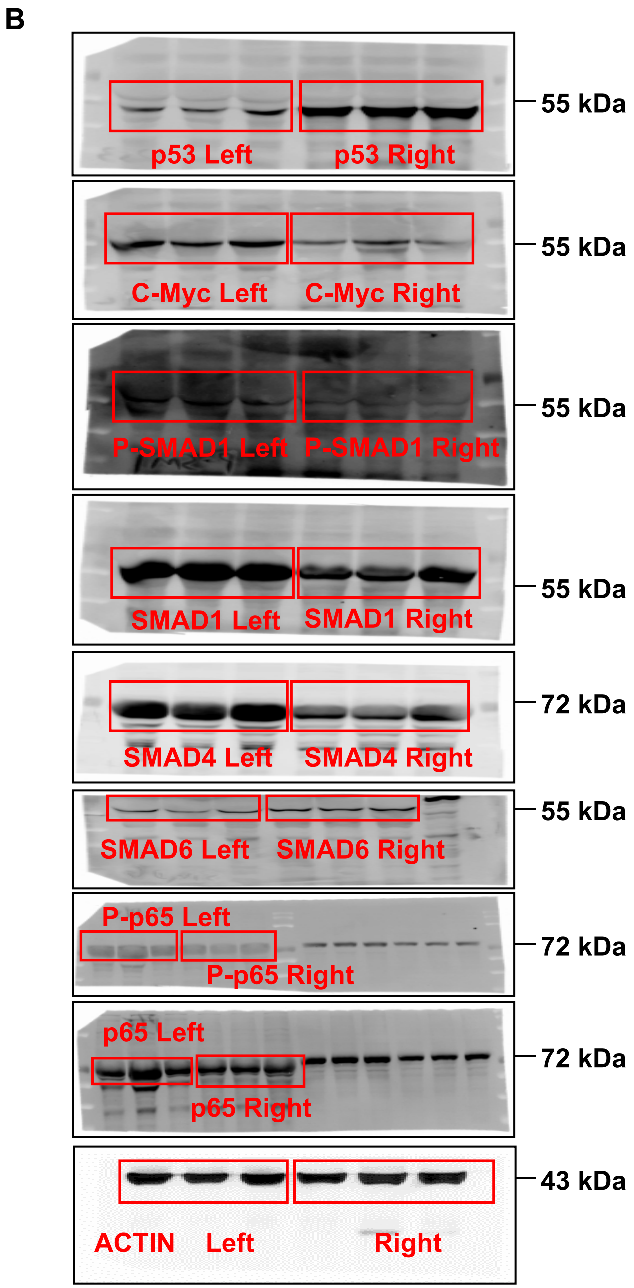
B
p53 Left
p53 Right
55 kDa
C-Myc Left
C-Myc Right
55 kDa
P-SMAD1 Left
P-SMAD1 Right
55 kDa
SMAD1 Left
SMAD1 Right
55 kDa
SMAD4 Left
SMAD4 Right
72 kDa
SMAD6 Left
SMAD6 Right
55 kDa
P-p65 Left
P-p65 Right
72 kDa
p65 Left
p65 Right
72 kDa
ACTIN
Left
Right
43 kDa

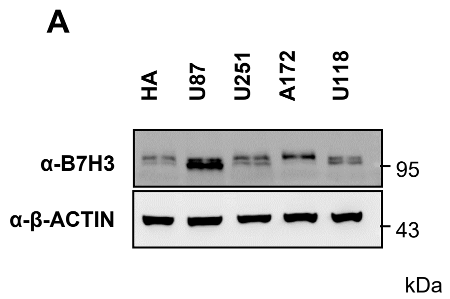

Supplementary Figure 1A

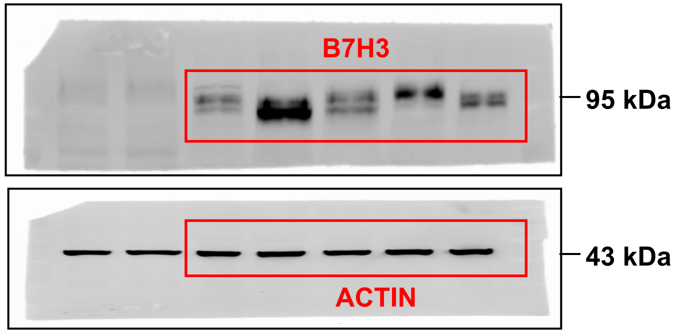

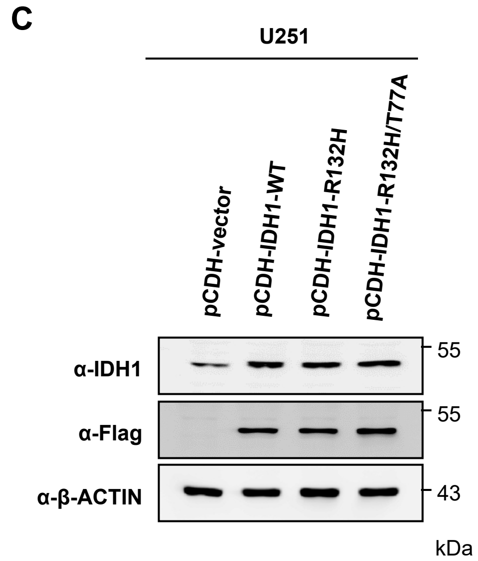

Supplementary Figure 2C

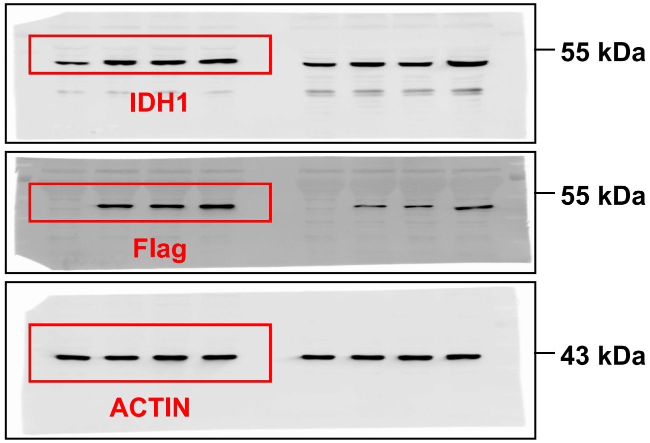

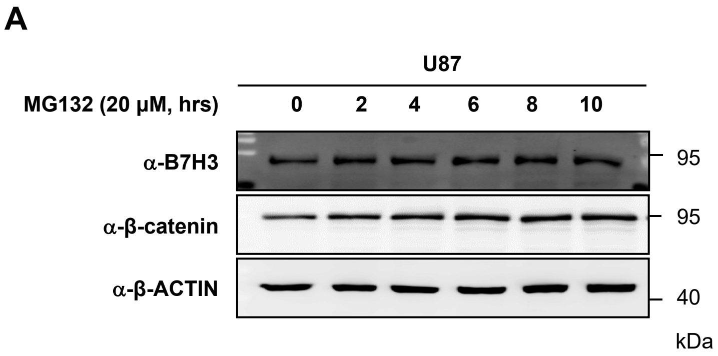

Supplementary Figure 3A

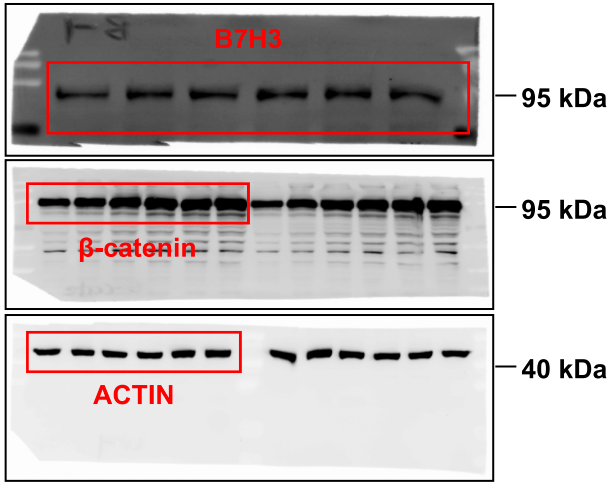

B

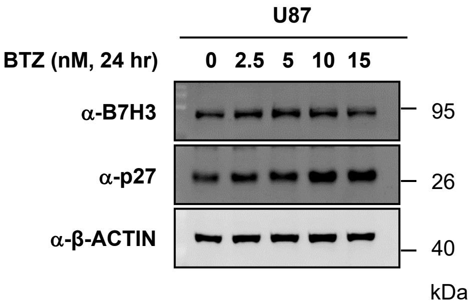

C

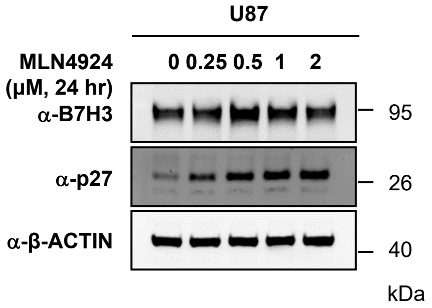

Supplementary Figure 3B

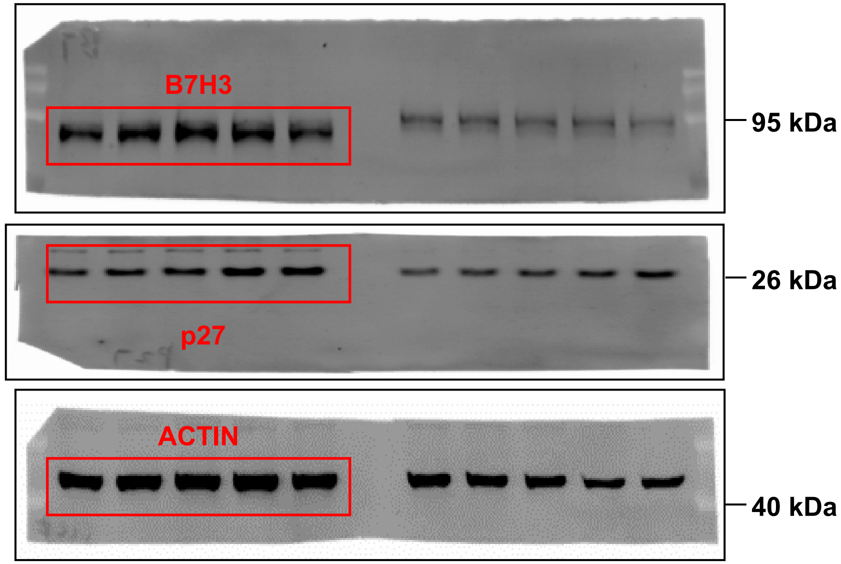

Supplementary Figure 3C

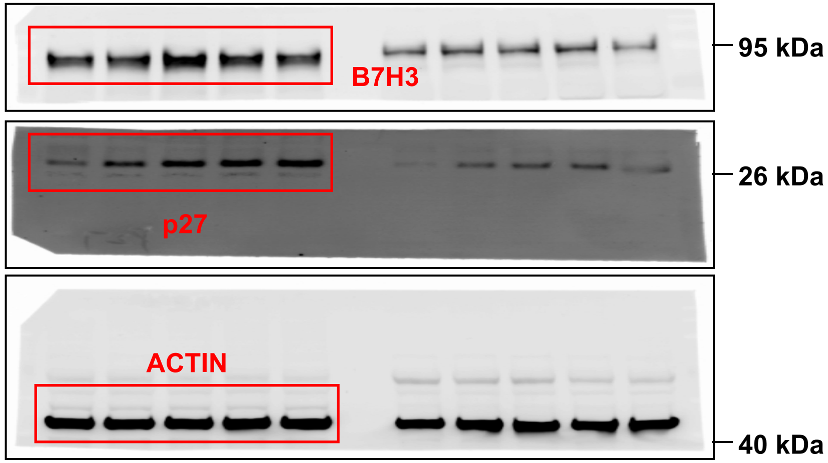

Supplementary Figure 4A-D

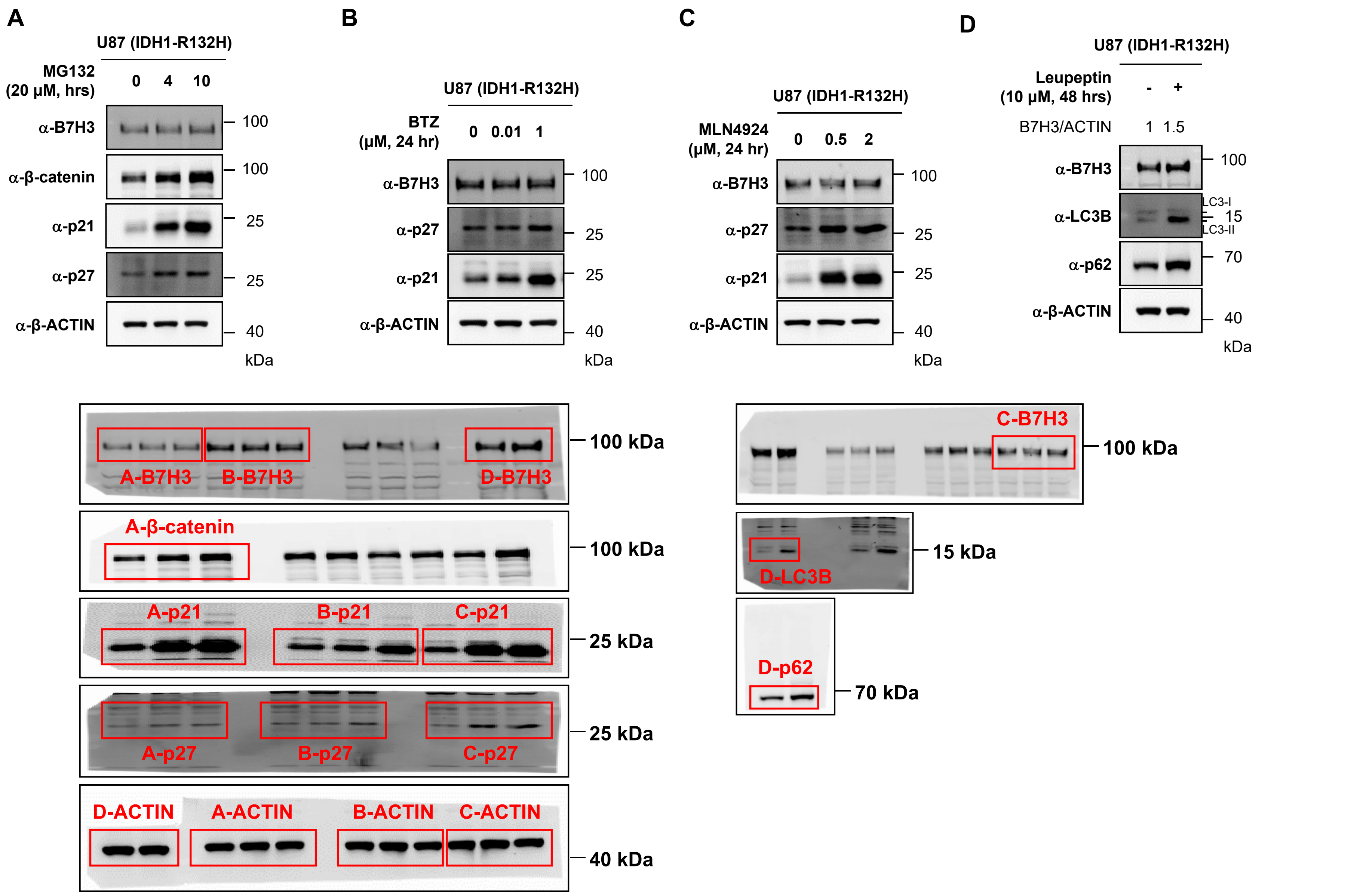

A: Supplementary Figure 4A **B:** Supplementary Figure 4B **C:** Supplementary Figure 4C **D:** Supplementary Figure 4D

Supplementary Figure 4E

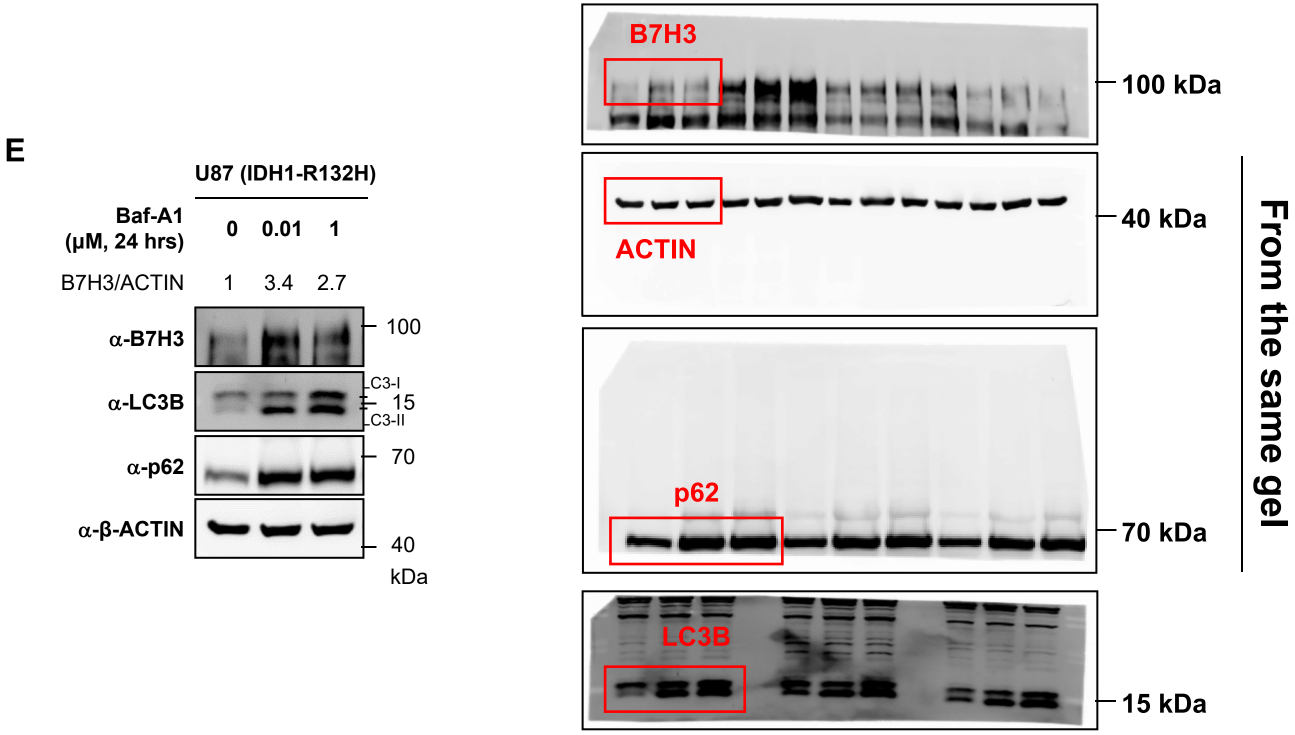

Supplement: Supplementary file 3 [file Data_Sheet_3.zip › Original data/WB original data-B7H3.pdf]
